# Sex and tissue resolved co-expression networks reveal a female placental–brain axis protective against prenatal PCB exposure

**DOI:** 10.1186/s13059-026-04052-8

**Published:** 2026-04-07

**Authors:** Kelly H. Chau, Kari Neier, Anthony E. Valenzuela, Rebecca J. Schmidt, Blythe Durbin-Johnson, Pamela J. Lein, Ian Korf, Janine M. LaSalle

**Affiliations:** 1 Department of Medical Microbiology and Immunology, School of Medicine, University of California, Davis, Davis, CA 95616 USA; 2 UC Davis Genome Center, University of California, Davis, Davis, CA 95616 USA; 3 MIND Institute, School of Medicine, University of California, Davis, Sacramento, CA 95817 USA; 4 Perinatal Origins of Disparities Center, University of California, Davis, Davis, CA 95616 USA; 5 Department of Molecular Biosciences, School of Veterinary Medicine, University of California, Davis, Davis, CA 95616 USA; 6 Department of Public Health Sciences, School of Medicine, University of California, Davis, Davis, CA 95616 USA; 7 Department of Molecular and Cellular Biology, University of California, Davis, Davis, CA 95616 USA

**Keywords:** Placental-brain axis, Neurodevelopmental disorders, Polychlorinated biphenyls, Folic acid interactions, Weighted gene correlation network, Differential gene expression, X chromosome, *Xist*

## Abstract

**Background:**

Neurodevelopmental disorders have a strong male bias that is poorly understood. The placenta provides molecular information about environmental interactions with genetics (including biological sex) that shape developmental processes in the brain. We investigate placental-brain transcriptional responses in an established mouse model of prenatal exposure to a human-relevant mixture of polychlorinated biphenyls (PCBs).

**Results:**

To understand sex, tissue, and dosage effects in embryonic (E18) brain and placenta RNAseq data, we use weighted gene correlation network analysis (WGCNA) to create gene networks that could be compared across sex or tissue. WGCNA reveals that expression within most correlated gene networks is significantly and strongly associated with PCB exposure, but frequently in opposite directions between male–female and placenta-brain comparisons. In WGCNA and differentially expressed gene analyses, more transcriptional changes are observed in male brain than placenta, but the reverse is seen in females. Furthermore, female X-inactive specific transcript (*Xist*) levels correlate with sex-specific and non-monotonic PCB dose response, suggesting an X-linked protective epigenetic mechanism. The transcriptomic effects of low-dose PCB exposure are significantly opposed by dietary folic acid supplementation across both sexes but are strongest in female placentas. PCB and folic acid interacting gene networks are enriched in metabolic pathways involved in energy usage and translation, with female-specific protective effects enriched in PPAR, thermogenesis, glycerolipid, and O-glycan biosynthesis, as opposed to toxicant responses in male brain.

**Conclusions:**

A female protective effect in response to prenatal PCB exposure appears to be mediated by dose-dependent sex differences in transcriptional modulation of placental metabolic pathways.

**Supplementary Information:**

The online version contains supplementary material available at 10.1186/s13059-026-04052-8.

## Background

The placenta is a unique tissue derived from both fetal trophectoderm and maternal decidua, but by mid-late gestation, fetal trophoblasts make up the majority of placental cell types [1, 2]. Placenta plays a critical role in the development of eutherian mammals by facilitating the exchange of nutrients, oxygen, and waste products, as well as providing protection from external environmental factors at the interface between the mother and fetus. In humans, term placenta is accessible as a birth byproduct and potentially a rich source of signature gene expression biomarkers that can provide important molecular clues concerning how the developing brain is impacted by in utero exposures through a shared “placental-brain axis”. The placental-brain axis could also be useful in investigating gene-by-environment interactions contributing to the perplexing increase in the prevalence of neurodevelopmental disorders (NDDs) of 1 in 5 males and 1 in 8 females in the US (2018–2021) [3].

Endocrine disrupting chemicals, including polychlorinated biphenyls (PCBs) are posited to be risk factors for male-biased autism spectrum disorders (ASDs) and other NDDs [4, 5]. In the NDD enriched-risk human prospective study “Markers of Autism Risks in Babies—Learning Early Signs” (MARBLES), detectable concentrations of PCBs in maternal blood were associated with adverse neurodevelopmental outcomes including ASD and with DNA methylation changes in known ASD risk genes [6–8]. Humans, including pregnant women and women of child-bearing age, are exposed to PCBs through inhalation and consumption of contaminated food and water, and the risk is particularly prominent if exposures are experienced in utero [9–11]. Interestingly, recent studies have shown that folic acid supplementation during pregnancy is associated with a reduced likelihood of ASD in offspring, suggesting a protective effect against environmental exposures [12–15]. This motivates investigation of folic acid’s protective effects in the presence of known neurodevelopmental toxicants such as PCBs.

In a mouse model of prenatal exposure to a mixture of PCB congeners detected in the MARBLES cohort, there were significant deficits in sociability and ultrasonic vocalizations, as well as enhanced repetitive behavior but only in males, specifically at the lowest dose tested (0.1 mg/kg/d) [16]. This observed sexual dimorphism, specifically a male bias in a multitude of behavioral phenotypes, raises the question of a “female protective effect” during PCB exposures experienced in utero that has been previously suggested in both genetic and environmental causes of NDDs [17–20]. Furthermore, the non-monotonic dose response of behavioral phenotypes manifesting only at the low dose, compared with vehicle, 1 mg/kg, or 6 mg/kg also remains unclear [16]. A whole-genome DNA methylation approach confirmed sex differences in DNA methylation profiles and revealed that the placenta and fetal brain share an NDD DNA methylation profile in a mouse model of prenatal PCB exposure [21]. These findings prompted investigation of the complex relationships between placental-brain axis, sexual dimorphism, and non-monotonic dosage effects in the context of prenatal exposure to PCBs.

To investigate possible sex differences in placental-brain transcriptomes, including effects of PCB dosage and folic acid supplementation, we exposed C57BL/6J females to varying doses (0, 0.1, 1, or 6 mg/kg) of a human-relevant PCB mixture representative of the PCB profiles in the human cohort MARBLES study. These experimental conditions were chosen to be consistent with the prior mice behavioral study of the same model [16]. To test whether folic acid supplementation during pregnancy could counteract the effects of prenatal exposure to PCBs, the diet was enriched with or without supplemental folic acid prior to timed mating. At embryonic day 18, whole brain and placenta were collected for RNA-sequencing. This mouse model was designed to identify tissue- and sex- specific gene expression signatures underlying the sexual dimorphism and non-monotonic dose response of the developmental neurotoxicity of PCBs, and the potential counteracting effects of folic acid during pregnancy.

## Results

### Experimental design to examine the dosage effects of a human-relevant PCB mixture and the potential protective effects of folic acid supplementation

**Fig. 1:**
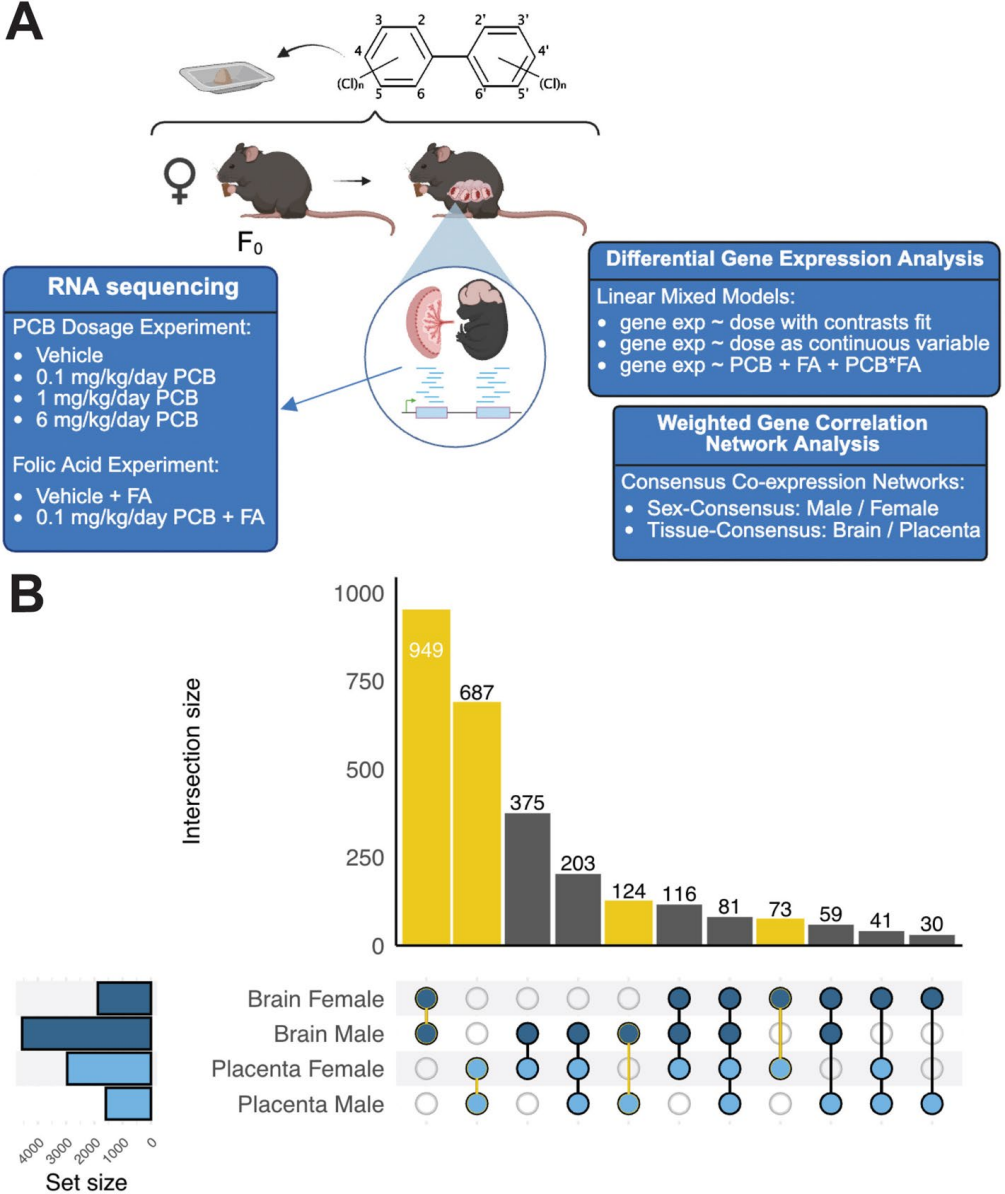
Experimental design and overlap of PCB dose-dependent differentially expressed genes (DEGs) by sex and tissue.** A** The MARBLES mixture of PCBs was given orally at daily doses preconception and prenatally until embryonic day 18 (E18) when placenta and fetal brain samples were harvested for RNAseq. There were two different experiments (PCB dosage and folic acid effect) and two different analysis approaches (DEG and WGCNA). **B** UpSet plot of DEGs (uncorrected p < 0.05) identified on the basis of PCB dose effects as a continuous variable. Overlaps between pairwise sample comparisons by matched tissue or sex are represented by yellow bars. The greatest number of PCB-associated DEGs were observed in the male brain, but more than twice as many DEGs overlapped between the male brain and the female placenta than between the male brain and the male placenta

Figure 1A shows the experimental design and treatment groups that were included to test the hypothesis that PCB-induced transcriptional changes in the placental-brain axis are affected by both dosage and sex. Dams were dosed daily for 2 weeks before conception, through breeding and gestation, until embryonic day 18 (E18). E18 in mice corresponds to a late‑embryonic stage of brain and placental development, analogous to mid‑gestation in humans. It is one timepoint along a broader developmental trajectory during which environmental influences can shape gene regulation. Concurrently, the placenta at E18 is a relevant time point to the human term placenta, having reached functional maturity and establishment of maternal–fetal exchange systems. Assessing matched fetal whole brain and placenta at E18 thus provides a valuable opportunity to investigate how term placenta can be an informative tissue source that can reflect environmental influences on neurodevelopment. The effect of dietary supplementation with folic acid was also investigated at a single low dose (0.1 mg/kg/d) of the PCB mixture because this was the dose at which most phenotypes were observed in the prior behavioral study [16]. RNA was isolated from E18 matched placenta and brain samples for transcriptome analyses via RNAseq (Quality control metrics are in Additional File 1). We then used two complementary bioinformatic approaches to analyze the RNAseq data. Traditional differentially expressed gene (DEG) analysis detects changes in individual genes in response to treatment and assumes that a small proportion of genes will change. In contrast, weighted gene correlation network analysis (WGCNA) is based on the biological principle that genes respond within correlated networks and pathways, thereby allowing pathway-focused rather than gene-focused analyses [22].

### Analysis of individual differentially expressed genes reveals complex sex, dosage, and tissue effects of PCB mixtures and folic acid

We first analyzed individually differentially expressed genes via limmaVoom, a statistical pipeline designed for RNA-seq data with multiple treatment groups. The results are summarized in Table 1 and Fig. 1B, which show strong effects of dosage, sex, and tissue type on the number of DEGs. For example, the number of DEGs followed a primarily monotonic dose–response pattern in males, with the most differentially expressed genes (DEGs) detected at the 6 mg/kg/day dose level vs. the vehicle control (318 genes) and when the PCB dose was modeled as a continuous variable (368 genes) (Table 1). In contrast, the largest number of DEGs from female brain was in the low dose (0.1 mg/kg/day) versus high dose (6 mg/kg/day) comparison (126 genes), with no significant DEGs in the vehicle vs. the highest dose group and only five DEGs when PCBs were modeled as a continuous variable. Like in the brain, differences in the number of placental DEGs were predominantly monotonic in males, but non-monotonic in females. Unlike in the brain, male placental DEGs were found in both the medium- and high-dose groups versus the vehicle group (393 and 360, respectively; Table 1). To find overlapping DEGs across tissue type and sex, an UpSet plot of DEGs (uncorrected *p* < 0.05) was generated, which shows the number of overlapping DEGs for the dose-responsive model (Fig. 1B). While matched tissues showed the greatest overlap regardless of sex, the male brain shared many more PCB DEGs with the female placenta (375 genes) than with the male placenta (124). The female brain was an outlier in showing the lowest overlap of PCB DEGs with other tissues. In agreement with these results, analyses of the global transcriptional variance by clustering, percent variance explained, and principal components analysis showed that sex was the largest component effect, followed by tissue (Additional file 2: Fig. S1 and S2). Specific examples of PCB dose-responsive DEGs showing sex-specific and non-monotonic effects are in Additional file 2: Fig. S3).

**Table 1:** Number of differentially expressed genes (DEG) by pairwise PCB dose comparisons or PCB dose as a continuous variable

Tissue	Sex	Comparison	Number of DEGs (FDR < 0.05)	Number Dose–Response DEGs (FDR < 0.05)
Brain	Females	Vehicle vs. 0.1 mg/kg/d	0	5
		Vehicle vs. 1 mg/kg/d	0	
		Vehicle vs. 6 mg/kg/d	0	
		0.1 mg/kg/d vs. 1 mg/kg/d	11	
		0.1 mg/kg/d vs. 6 mg/kg/d	126	
		1 mg/kg/d vs. 6 mg/kg/d	1	
	Males	Vehicle vs. 0.1 mg/kg/d	0	368
		Vehicle vs. 1 mg/kg/d	0	
		Vehicle vs. 6 mg/kg/d	318	
		0.1 mg/kg/d vs. 1 mg/kg/d	11	
		0.1 mg/kg/d vs. 6 mg/kg/d	82	
		1 mg/kg/d vs. 6 mg/kg/d	62	
Placenta	Females	Vehicle vs. 0.1 mg/kg/d	190	25
		Vehicle vs. 1 mg/kg/d	37	
		Vehicle vs. 6 mg/kg/d	178	
		0.1 mg/kg/d vs. 1 mg/kg/d	8	
		0.1 mg/kg/d vs. 6 mg/kg/d	124	
		1 mg/kg/d vs. 6 mg/kg/d	2	
	Males	Vehicle vs. 0.1 mg/kg/d	0	3
		Vehicle vs. 1 mg/kg/d	393	
		Vehicle vs. 6 mg/kg/d	360	
		0.1 mg/kg/d vs. 1 mg/kg/d	233	
		0.1 mg/kg/d vs. 6 mg/kg/d	393	
		1 mg/kg/d vs. 6 mg/kg/d	0	

A secondary objective of this study was to investigate whether dietary FA supplementation during development could counteract the effects of developmental PCB exposure. Therefore, we modeled the interaction effect of low dose PCB (0.1 mg/kg/d) and dietary FA supplementation to identify DEGs with significant effects of FA on PCB exposure. Only the female placenta had any DEGs whose FA had a significant effect on PCB (18; Table 2).

**Table 2:** Number of differentially expressed genes (DEG) by pairwise comparisons of low dose PCBs with or without folic acid (FA) supplementation

Tissue	Sex	Comparison	Number of DEGs (FDR < 0.05)	Number of interxn DEGs (FDR < 0.05)
Brain	Females	Vehicle vs. Vehicle + FA	0	0
		Vehicle vs. 0.1 mg/kg/d	0	
		Vehicle vs. 0.1 mg/kg/d + FA	1	
		Vehicle + FA vs. 0.1 mg/kg/d	1	
		Vehicle + FA vs. 0.1 mg/kg/d + FA	0	
		0.1 mg/kg/d vs. 0.1 mg/kg/d + FA	9	
	Males	Vehicle vs. Vehicle + FA	0	0
		Vehicle vs. 0.1 mg/kg/d	0	
		Vehicle vs. 0.1 mg/kg/d + FA	1	
		Vehicle + FA vs. 0.1 mg/kg/d	0	
		Vehicle + FA vs. 0.1 mg/kg/d + FA	1	
		0.1 mg/kg/d vs. 0.1 mg/kg/d + FA	4	
Placenta	Females	Vehicle vs. Vehicle + FA	18	18
		Vehicle vs. 0.1 mg/kg/d	1	
		Vehicle vs. 0.1 mg/kg/d + FA	47	
		Vehicle + FA vs. 0.1 mg/kg/d	0	
		Vehicle + FA vs. 0.1 mg/kg/d + FA	6	
		0.1 mg/kg/d vs. 0.1 mg/kg/d + FA	15	
	Males	Vehicle vs. Vehicle + FA	0	0
		Vehicle vs. 0.1 mg/kg/d	0	
		Vehicle vs. 0.1 mg/kg/d + FA	12	
		Vehicle + FA vs. 0.1 mg/kg/d	0	
		Vehicle + FA vs. 0.1 mg/kg/d + FA	0	
		0.1 mg/kg/d vs. 0.1 mg/kg/d + FA	0	

### Experimental design of consensus modules within weighted gene correlation network analyses

Because of the complex relationships observed by sex, PCB dosage, and tissue type from the DEG analyses, we expanded on the analyses to use a systems-based approach to comprehensively understand each of these variables in the transcriptional response to the prenatal PCB mixture. Weighted gene correlation network analysis (WGCNA) is a method to perform pairwise correlations among genes that allows grouping of co-expressed groups of genes into modules [22]. Each module is then represented by an eigengene value as a reduced representative of the expression levels of all the genes within a module. We are then able to examine the expression dynamics of groups of genes and gene pathways associated with each PCB dose and/or FA supplementation, comparing the same groups of genes across the brain-placenta axis and across both sexes.

First, we used a sex-consensus co-expression network of gene expression profiles across males and females, and then stratified the data via these sex-consensus modules to investigate sex-specific impacts on the brain versus placenta following prenatal exposure to PCBs (0, 0.1, 1, or 6 mg/kg/d). Twenty-three consensus modules between males and females were defined for the brain, with an overall preservation score of 0.57, whereas a separate set of 23 consensus modules between males and females was defined for the placenta, with an overall preservation score of 0.60 (Additional File 2: Fig. S4-S5; module genes and hubs in Additional File 3: Tables S1-S2), both reflecting moderate correlations in transcriptomes between the sexes.

Second, we used a tissue-consensus co-expression network of gene expression profiles across the brain and placenta and then stratified the data via these tissue-consensus modules to investigate tissue-specific PCB effects in males versus females. Twenty-one consensus modules between the brain and placenta were defined for males, with an overall preservation score of 0.68, whereas a separate set of 18 consensus modules between the brain and placenta was defined for females, with an overall preservation score of 0.75 (Additional File 2: Fig. S6-S7; module genes and hubs in Additional File 3: Tables S3-S4), both reflecting moderate correlations in transcriptomes between the tissues.

**Fig. 2:**
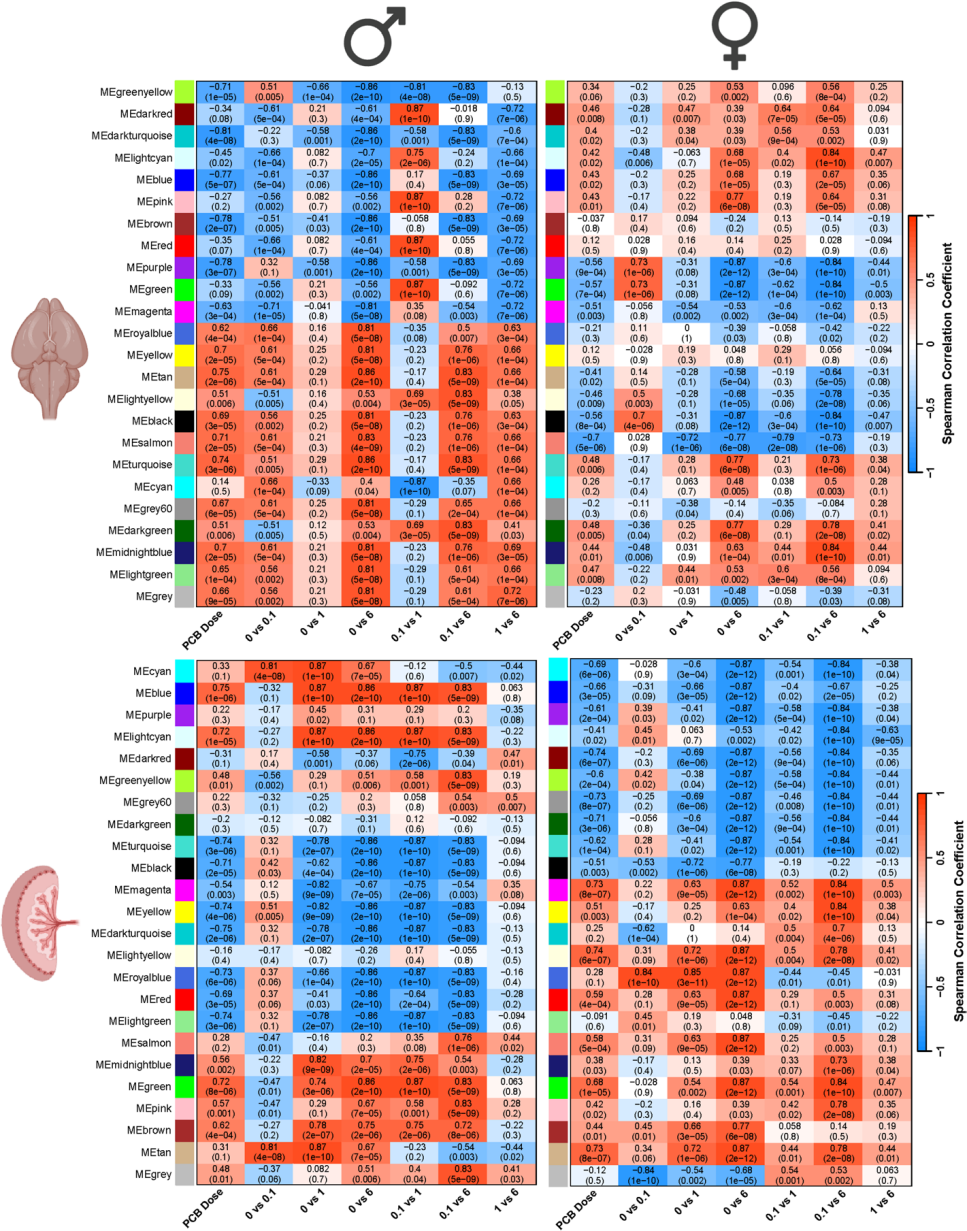
Sex-consensus modules of the brain and placenta reveal broad transcriptional impacts with sex-specific and non-monotonic dosage effects in response to prenatal PCB exposure. Two different sets of WGCNA modules (y-axes) were defined for either the brain (top) or placenta (bottom) that could be compared across sexes (left–right comparison of the same module). Spearman correlations with each PCB dose comparison (x-axes) are color coded (red, positive; blue, negative) with both the r value of correlation and the p value provided for each module and trait

**Fig. 3:**
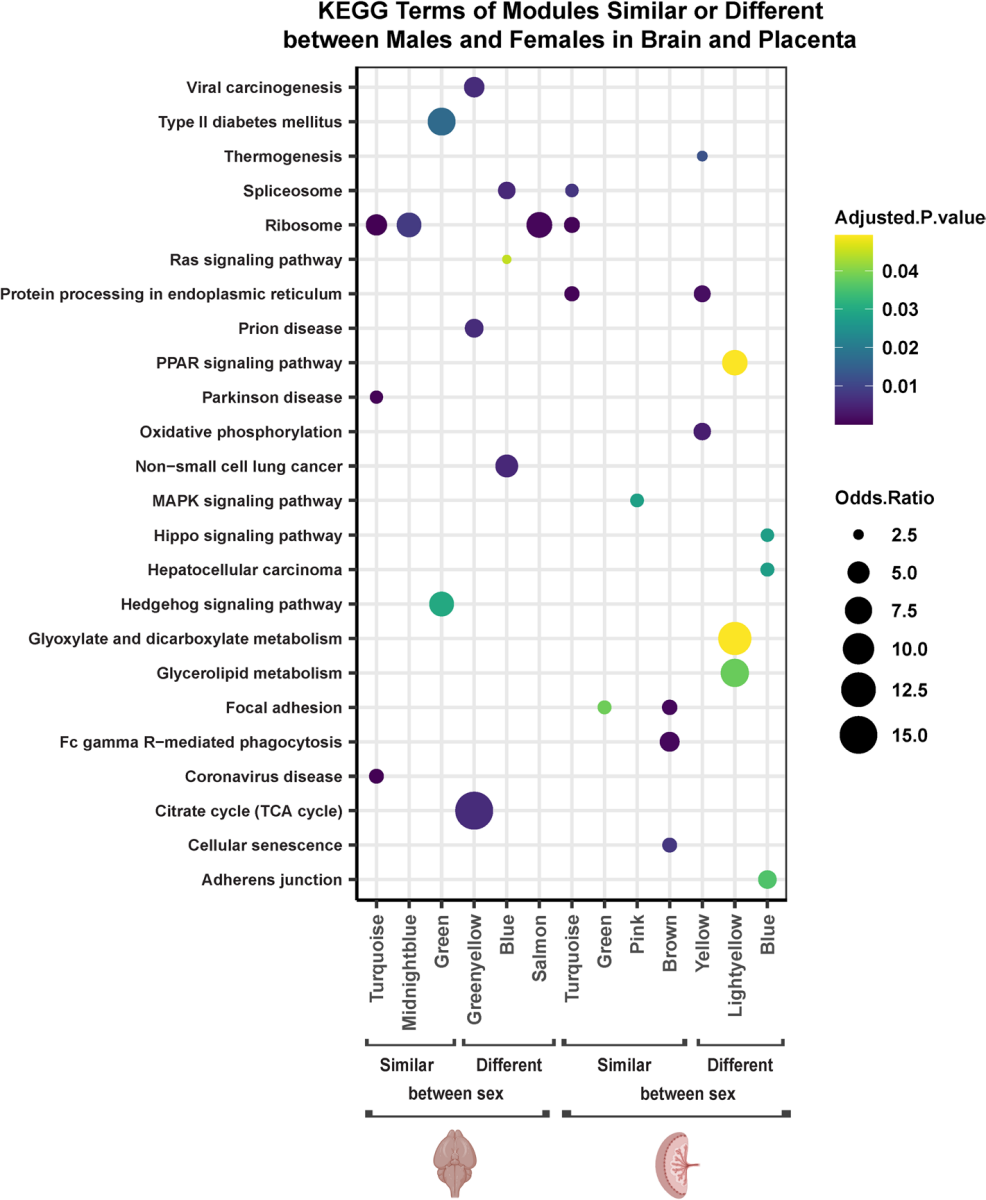
Significantly enriched KEGG gene pathways for sex-consensus modules with significant PCB associations in similar vs. different directions between males and females. Modules that had correlations in the same direction between sexes in at least 5 columns in Fig. 2 heatmap were considered similar between the sexes. Modules with correlations in the opposite direction between sexes in at least 5 columns were considered different between sexes. The top 3 KEGG terms enriched in these modules, along with their p-values and log odds ratios are shown (FDR adjusted p-values of 0.05 were considered statistically significant)

**Fig. 4:**
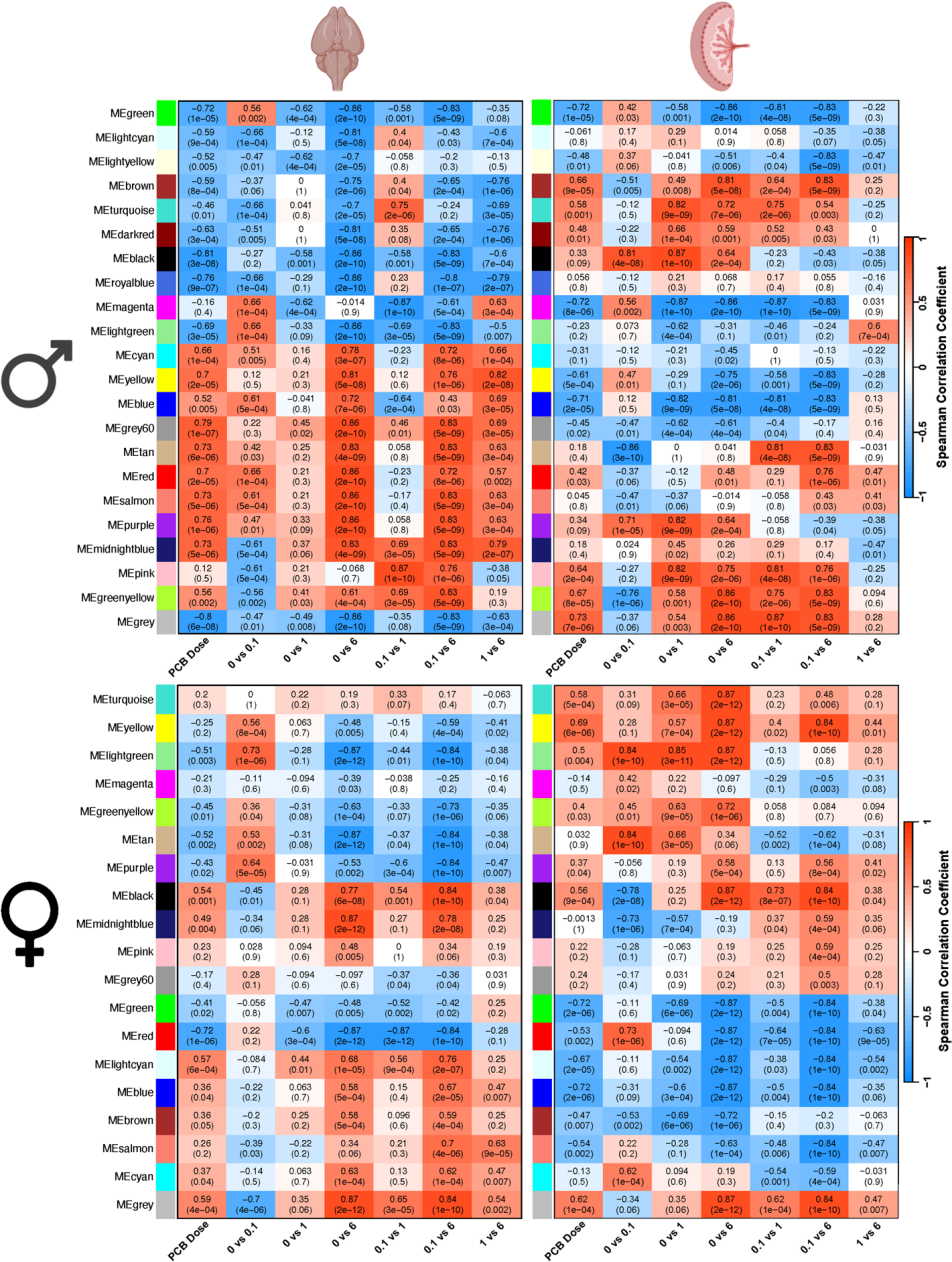
Tissue-consensus modules of the brain and placenta reveal broad transcriptional impacts with sex-specific effects in response to prenatal PCB exposure. Two different sets of WGCNA modules (y-axes) were defined for either males (top) or females (bottom) that could be compared across the brain and placenta (left–right comparison of the same module). Spearman correlations with each PCB dose comparison (x-axes) are color coded (red, positive; blue, negative) with both the r value of correlation and the p value provided for each module and trait

**Fig. 5:**
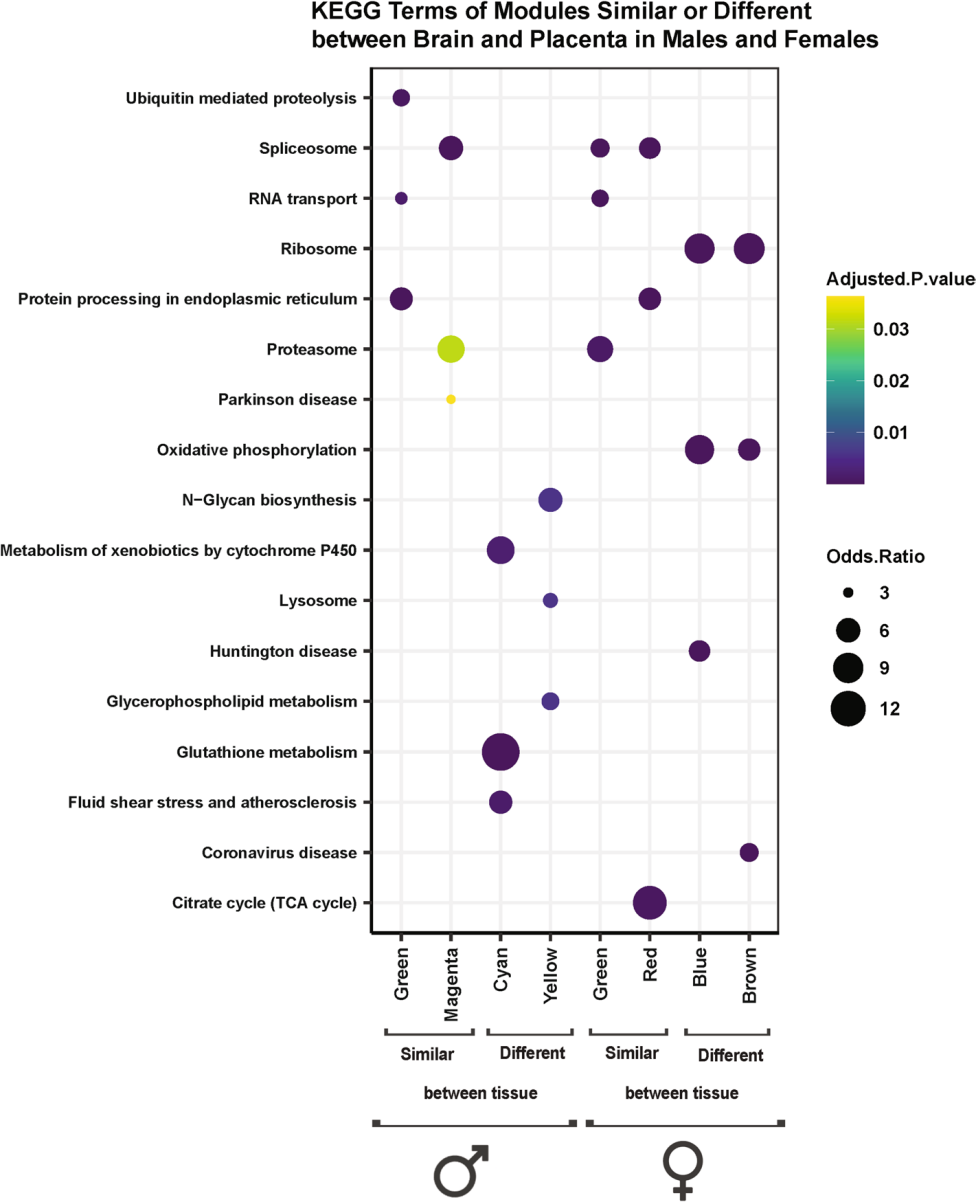
Significantly enriched KEGG gene pathways for tissue-consensus modules with significant PCB associations in similar vs. different directions between the brain and placenta. Modules that had correlations in the same direction between tissues in at least 5 columns in Fig. 4 heatmap were considered similar between tissues. Modules with correlations in the opposite direction between tissues in at least 5 columns were considered different between tissues. The top 3 KEGG terms enriched in these modules, along with their p-values and log odds ratios are shown (FDR adjusted p-values of 0.05 were considered statistically significant)

**Fig. 6:**
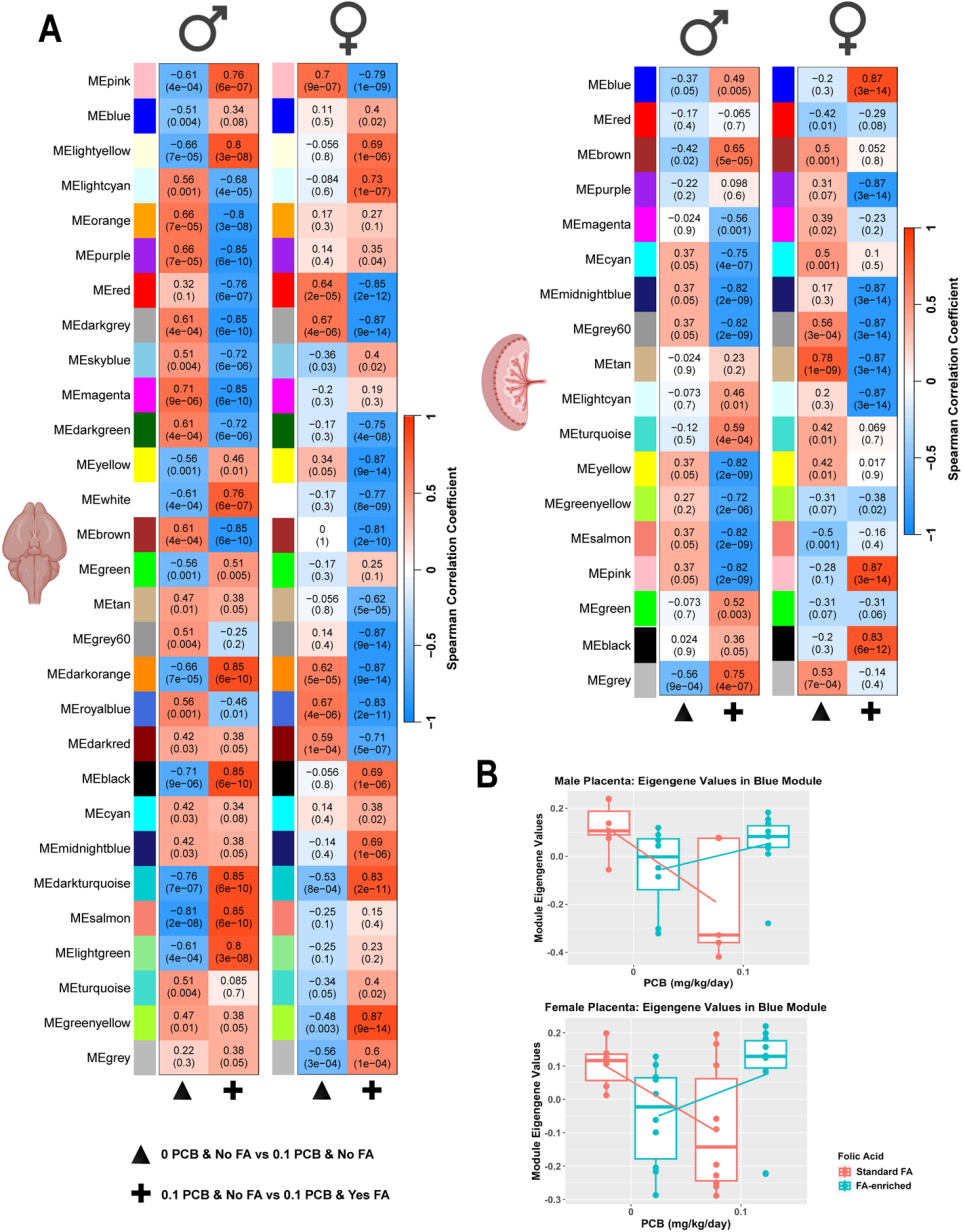
Sex-consensus modules of the brain and placenta reveal broad transcriptional impacts with sex-specific counteracting effects of folic acid supplementation on prenatal PCB exposure.** A** Two different sets of WGCNA modules (y-axes) were defined for either the brain (left) or placenta (right) that could be compared across sexes (left–right sex comparison of the same module). Only low-dose PCB effects without (triangle) or with (+) folic acid (FA) supplementation are shown here; additional pairwise comparisons are shown in Additional file 2: Fig. S12. Spearman correlations with each PCB dose comparison (x-axes) are color coded (red, positive; blue, negative) with both r values of correlation and p values provided for each module and trait. **B** Two examples of significant reversal effects are plotted for the Blue module that showed the same effects in male and female placentas

**Fig. 7:**
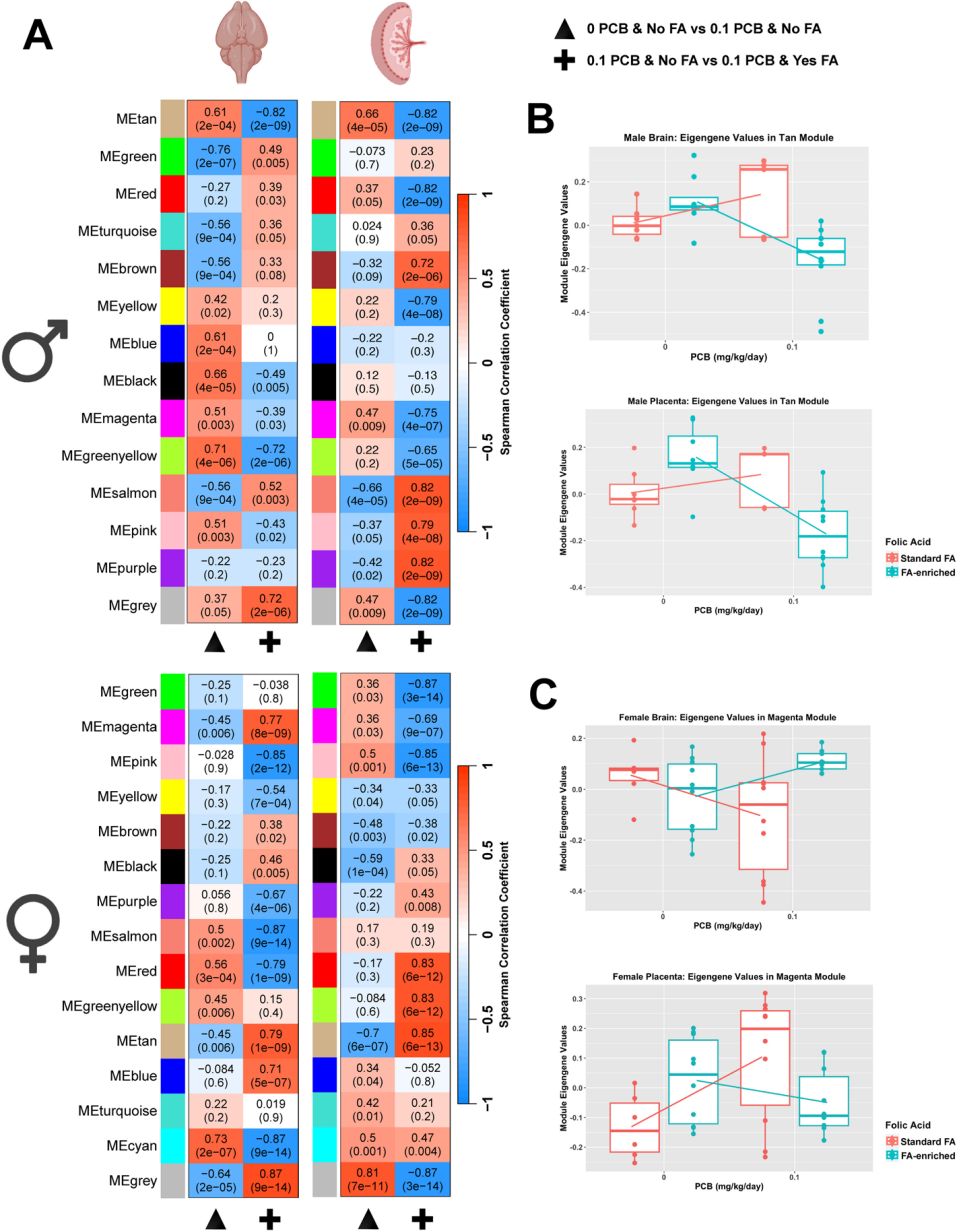
Tissue-consensus modules of the brain and placenta reveal broad transcriptional impacts with sex-specific counteracting effects of folic acid supplementation on prenatal PCB exposure.** A** Two different sets of WGCNA modules (y-axes) were defined for either males (top) or females (bottom) that could be compared across sexes (left–right sex comparison of the same module). Only low-dose PCB effects without (triangle) or with (+) folic acid (FA) supplementation are shown here, additional pairwise comparisons are shown in Additional file 2: Fig. S13. Spearman correlations with each PCB dose comparison (x-axes) are color coded (red, positive; blue, negative) with both r values of correlation and p values provided for each module and trait. **B** Examples of significant reversal effects are plotted for the Tan module, which showed the same effects in the placenta and brain. **C** Examples of opposite FA effects are plotted for the Magenta module

**Fig. 8:**
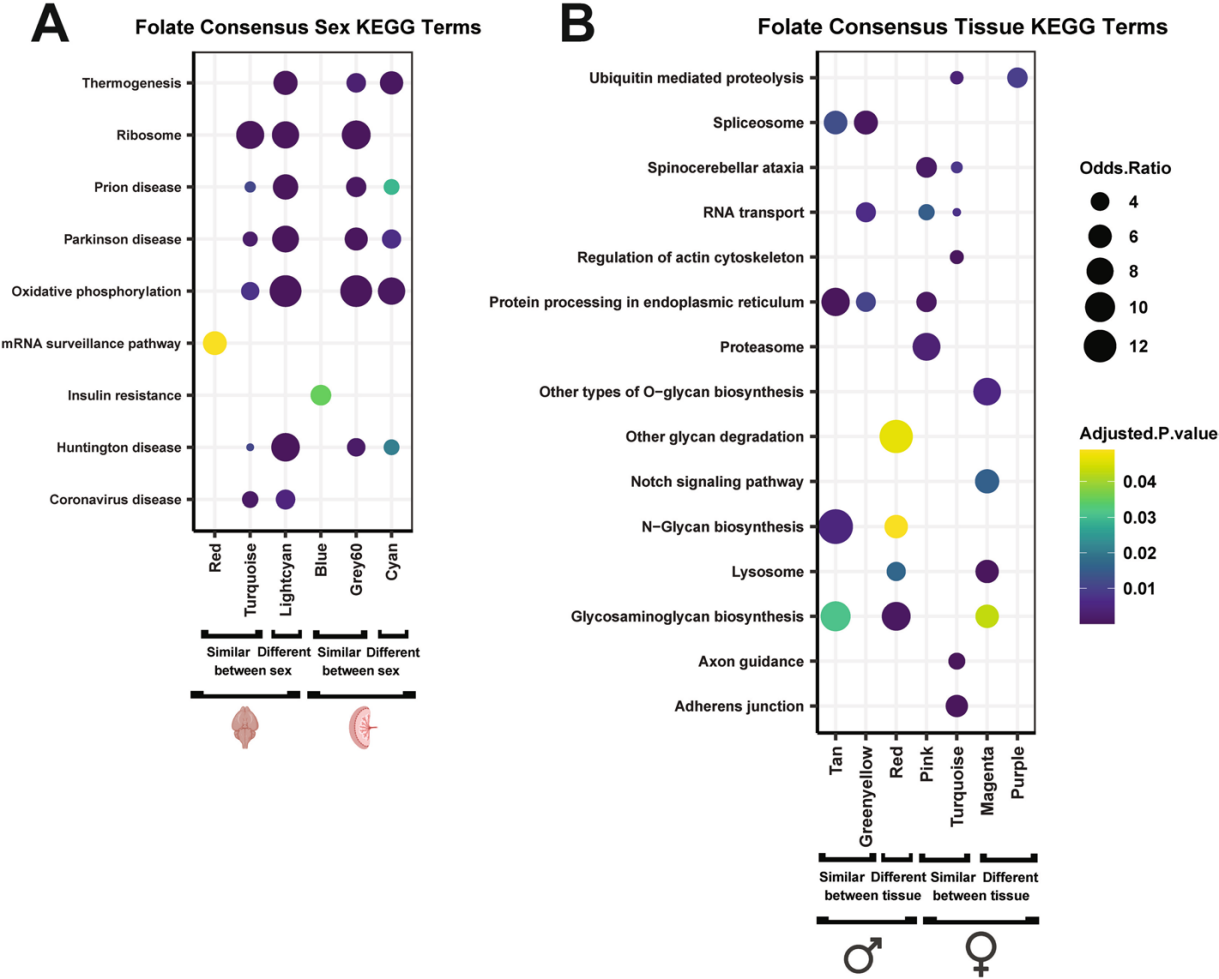
Significantly enriched KEGG gene pathways for folic acid + low dose PCB sex- and tissue-consensus modules. Modules that had correlations in the same direction between sex or tissues in at least 5 columns in Figs. 6 and 7 heatmaps were considered similar between tissues. Modules with correlations in the opposite direction between tissues in at least 5 columns were considered different between tissues. The top 3 KEGG terms enriched in these modules, along with their p-values and log odds ratios are shown (FDR adjusted p-values of 0.05 were considered statistically significant)

**Fig. 9:**
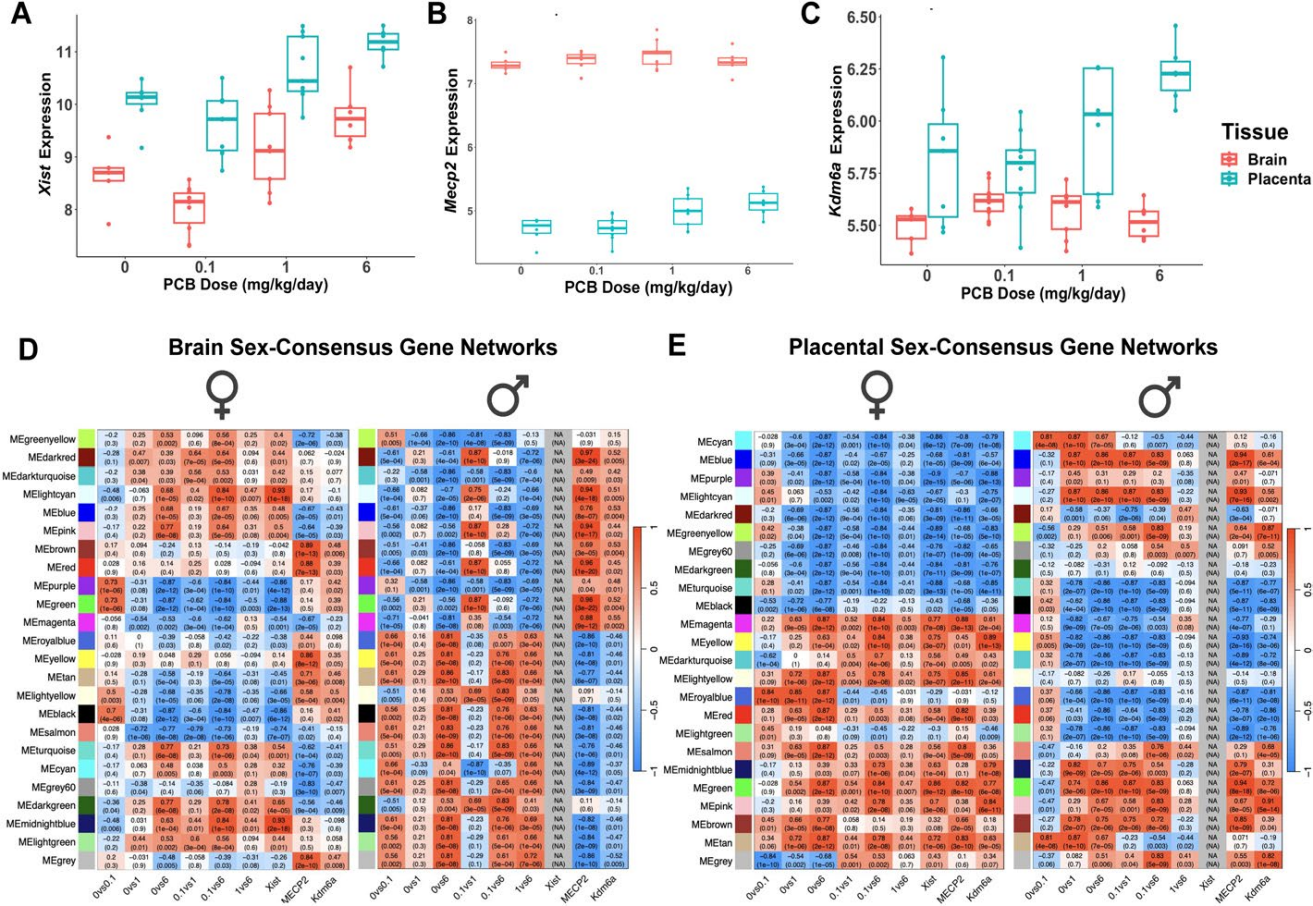
Placental and brain *Xist* levels respond to PCB dosage and correlate with transcriptional sex differences. Three X-linked transcripts encoding epigenetic regulators are plotted for PCB dosage effects in female brain and placenta (**A**-**C**). **A** *Xist*, a female-specific lncRNA was a PCB DEG and shows a non-monotonic dosage effect in both brain and placenta. **B** *Mecp2*, subject to X chromosome inactivation, is not affected by PCB dose and is expressed higher in brain than placenta. **C** *Kdm6a* that escapes X chromosome inactivation is a PCB dosage DEG in placenta but not brain. **D-E** The same heatmaps shown in Fig. 2 of sex-consensus modules but with correlations for the individual genes *Xist*, *Mecp2*, and *Kdm6a* shown to the right. Because males have negligible levels of *Xist*, that row is grey. In female brain and placenta, *Xist* is highly correlated with module eigenmode vales showing sex differences

Finally, we constructed networks for the same two module types (sex-consensus and tissue-consensus) but only for samples relevant to the FA supplementation experiment (vehicle, vehicle + FA, 0.1 mg/kg/d PCBs, 0.1 mg/kg/d PCBs) (Additional File 2: Fig. S8-S11; module genes and hubs in Additional File 3: Tables S5-S8). For each of these analyses, the modules are arbitrarily named by color. However, since these color names are reassigned in each separate module set, the same color can represent a different group of genes in different figure panels. Module colors compared left-to-right in figures always compare the same module, which represents the same group of genes (Figs. 2, 3, 4, 5, 6, 7, 8, 9).

### Sex-consensus modules of the brain and placenta reveal broad transcriptional impacts with sex-specific and non-monotonic dosage effects in response to prenatal PCB exposure

The sex-consensus modules allow the comparison of the same groups of correlated genes to be compared between males and females separately in the brain and placenta (Fig. 2, y-axes). The module eigengene values, reflecting the expression levels of all co-expressed genes within each module, were then correlated with PCB dosage effects, with most modules showing significant (*p* < 0.05) and strong (> 0.5 r^2^) positive (red) or negative (blue) associations with PCBs in at least one sex, tissue, or dosage. Considering PCB as a continuous variable (column 1 of x-axes), the male brain samples showed much stronger correlations across all modules than did the female brain samples, a result that was not observed in the placenta. Interestingly, several modules were strongly correlated with PCB dose in the same direction between males and females, whereas other modules were strongly correlated in the opposite direction between males and females (Table 3). Pairwise comparisons of dosage groups were further investigated to identify potential non-monotonic dosage effects. The most apparent overall non-monotonic effect was the dampening effect of vehicle (0) versus 1 mg/kg/day PCB, which was observed across most modules in the male brain, but not in the female brain or male placenta. These results indicate that the 0.1 and 6 mg/kg/day doses had greater transcriptomic effects than the 1 mg/kg/day dose did, but that this non-monotonic PCB effect was dependent on both sex and tissue.

**Table 3:** List of modules in the same or opposite directions across sex

	**Modules in similar directions** **between males and females**	**Modules in opposite directions between males and females**
**Brain**	MEpurple, MEmagenta, MEturquoise, MEdarkgreen, MEmidnightblue, MElightgreen	MEgreenyellow, MEdarkturquoise, MElightcyan, MEblue, MEroyalblue, MEtan, MElightyellow, MEblack, MEsalmon, MEgrey60, MEgrey
**Placenta**	MEdarkred, MEdarkgreen, MEturquoise, MEblack, MElightgreen, MEsalmon, MEmidnightblue, MEgreen, MEpink, MEbrown	MEblue, MEpurple, MElightcyan, MEgreenyellow, MEgrey60, MEmagenta, MEyellow, MEdarkturquoise, MElightyellow, MEred

For each of the sex consensus modules in the brain and placenta, genes within each of the modules were investigated for enrichment within known Gene Ontology (GO) and KEGG pathways. To examine potential gene pathways involved in sex-specific PCB effects, we focused on modules with significantly enriched KEGG pathway terms that are transcriptionally impacted by PCBs either in the same or different correlation directions between the sexes (Fig. 3). Ribosome was the most frequent KEGG term that was predominantly similarly impacted by PCBs in the brain and placenta of both sexes. Interestingly, ribosome functions were also enriched in the salmon module in the brain, which showed opposite correlations with PCB dose in male versus female brains, but included a distinct group of genes more specifically involved in mitochondrial translation by GO biological processes (Fig. 3, Supplementary Table 1). The citrate (TCA) pathway was highly enriched in the brain Greenyellow module, which was also correlated with PCB in opposite directions between the sexes. In the placenta, sex differences were observed in modules enriched for Oxidative phosphorylation, Hippo and PPAR signaling, as well as Glyoxylate and dicarboxylate and Glycerolipid metabolism (Fig. 3).

### Tissue-consensus modules reveal placenta-brain axis transcriptional differences between sexes enriched in ribosome and oxidative phosphorylation pathways

The tissue-consensus modules allow the comparison of the same groups of correlated genes to be compared between brain and placental separately in males and females (Fig. 4, y-axes). Considering PCB as a continuous variable (column 1 of x-axes), the male brain samples showed much stronger correlations across all modules than did the female brain samples, a result that was not observed in the placenta, and was consistent with the results from the sex-consensus modules (Fig. 2) and DEG analyses (Fig. 1B). While several modules are strongly correlated with PCB dose in the same direction between the brain and placenta, other modules are strongly correlated in the opposite direction between the brain and placenta (Table 4). Non-monotonic effects from pairwise analyses of PCB dosage were detected in most modules in the brain, but not in the placenta, and these effects were strongest in the male brain. The pairwise comparisons with the 1 mg/kg/day dose showed reduced correlations with module transcript levels specifically in the brain, similar to what was observed using sex-consensus modules. These results further confirm that non-monotonic transcriptional effects of PCBs are sex- and tissue-dependent.

**Table 4:** List of modules in the same or opposite directions across tissue

	**Modules in similar directions** **between brain and placenta**	**Modules in opposite directions between brain and placenta**
**Male**	MEgreen, MElightyellow, MEmagenta, MElightgreen, MEmidnightblue, MEpink, MEgreenyellow	MEbrown, MEdarkred, MEcyan, MEyellow, MEgrey60, MEgrey
**Female**	MEturquoise, MEmagenta, MEblack, MEgreen, MEred, MEgrey	MEyellow, MElightgreen, MEgreenyellow, MEpurple, MEgrey60, MElightcyan, Meblue, MEbrown, MEsalmon, MEcyan

To examine potential gene pathways involved in the tissue-specific PCB effects across both sexes, we focused on the modules with significantly enriched KEGG pathway terms that are transcriptionally impacted by PCBs either in the same or different correlation directions between the brain and placenta (Fig. 5). Similar to what was observed from sex-consensus modules, enriched KEGG terms were related to broad pathways in cellular metabolism and the diseases associated with their dysregulation. Interestingly, Ribosome and Oxidative Phosphorylation terms were uniquely enriched in females for two modules (blue and brown) that directionally differed in their transcriptional response between the brain and placenta, with a specific downregulation in the placenta. The Proteosome KEGG pathway (enriched in magenta in males and green in females) was similarly altered by PCBs in the brain and placenta across both sexes, but the TCA pathway was uniquely enriched in a female module (red in females) with similar brain-placenta transcriptional changes. The cyan module in males, enriched for Glutathione and Cytochrome P450 metabolism terms that are important in toxicant responses, was significantly increased in brain (0.1 and 6 mg/kg/d doses) but unchanged (0.1 and 1 mg/kg/d doses) or significantly decreased (6 mg/kg/d) in the placenta. These results suggest that the female placenta utilizes different metabolic responses to PCBs involving reduction of energy pathways, whereas the male placenta has a lower response to toxins than does the male brain.

### The low-dose PCB transcriptome effects were partially counteracted by folic acid dietary supplementation

To examine potential interaction effects between low dose PCB and dietary folic acid, we performed sex-consensus and tissue-consensus modules again but only for four groups (0 PCB & Standard FA, 0.1 mg/kg/d PCB & Standard FA, 0 PCB & FA-enrichment, 0.1 mg/kg/d PCB & FA-enrichment)(Additional File 2: Tables S8-S11). The heatmaps of all pairwise comparisons for module correlations (Additional File 2: Fig. S12-S13) revealed that the strongest evidence for interaction effects was for the comparisons of the PCB effect (0 PCB & Standard FA versus 0.1 PCB & Standard FA) and the PCB + FA-enrichment effect (0.1 PCB & Standard FA versus 0.1 PCB & FA-enrichment). These two comparisons are shown as heatmaps in Fig. 6 (sex-consensus FA) and Fig. 7 (tissue consensus FA) with the modules enriched for significant KEGG terms shown in Fig. 8.

The PCB-FA sex-consensus modules showed the expected strong associations with low dose PCB alone (column 1, triangle) in the male brain (Fig. 6A) as well as strong associations in the opposite direction in the presence of FA-enrichment. The female brain also showed the opposite direction of association with FA-enrichment + PCB even though the PCB effects alone were less prominent than those in the male brain. In the placenta, there were modules that were similar and others that were different between males and females, and the opposite effect was observed for FA-enrichment + PCB in the modules of both categories (Fig. 6B). The interaction effects of low dose PCB and FA are plotted for individual placental samples for the Blue sex-consensus module to show an example of the counteracting effect of FA-enrichment on the effect of PCB on transcript levels (Fig. 6C).

The PCB-FA tissue-consensus modules also showed opposing effects of FA in multiple modules, although in general the module associations with FA + PCB effects were stronger in the male placenta than in the brain (Fig. 7A). An example of an opposing but overcompensating effect is plotted for the PCB and FA interaction effect in the brain versus placenta for the tan module (Fig. 7B). The FA + PCB interaction effect was greater in females than in males, as the female transcriptome presented six modules with opposite directional FA + PCB associations between the brain and placenta, compared with only three in males (Fig. 7A). For example, the magenta tissue-consensus module in females showed increased transcript levels in the placenta but decreased levels in the brain in response to PCBs, but both effects were counteracted by FA-enrichment (Fig. 7C).

KEGG pathway enrichment analyses for PCB-FA sex-consensus modules revealed similar pathways that were observed for PCB dosage effects, including Ribosome, Oxidative phosphorylation, and diseases associated with these pathways, with the Thermogenesis pathway being more enriched in modules showing sex differences (Fig. 8A). The FA tissue-consensus modules that differed between the brain and placenta were uniquely enriched in Lysosome (male and female), Other glycan degradation (male only), Other types of O-glycan biosynthesis (female only), and Notch signaling (female only) (Fig. 8B). Together, these results demonstrate that the opposing effect of FA on PCB-induced transcriptional changes acts broadly through energy-regulating gene pathways, with some differences unique to the female placenta.

### Placental and brain *Xist* levels respond to PCB dosage and correlate with transcriptional sex differences

An inherent property of the sex-consensus gene modules shown in Fig. 2 is that they exclude transcripts with a strong sex bias, but we tested the hypothesis that three X-linked transcripts with epigenetic functions, *Xist*, *Mecp2*, and *Kdm6a*, could be independently associated with these correlated gene networks (Fig. 9, last 3 columns). *Xist* is expressed exclusively from the inactive X chromosome, *Kdm6a* escapes X chromosome inactivation, and *Mecp2* is subject to X chromosome inactivation [23, 24]. In female brain and placenta, the female-specific *Xist* long noncoding RNA was a significant differentially expressed gene (DEG) by PCB dosage, with increased expression at 1 and 6, but decreased expression at 0.1 compared to 0 mg/kg/day doses (Fig. 9A). In contrast, *Mecp2* was more highly expressed in brain than placenta, but showed no significant change with PCB exposure and was not a significant PCB DEG, while *Kdm6a* was a DEG elevated in response to PCB only in placenta, not brain (Fig. 9B-C). Across these mid and high PCB doses, brain *Xist* levels directionally correlated with the expression of gene modules that were in opposite directions between the sexes (Fig. 9D). Consistent with this finding, *Xist* was the hub gene of the black module from the female tissue consensus module that showed a similar non-monotonic PCB dose responses in both brain and placenta (Fig. 4, Table S4) and the tan module from the female PCB ± FA-enrichment network that showed a strong opposing effect in both tissues (Fig. 7A, Table S8). In contrast, both *Mecp2* and *Kdm6a* showed opposite transcriptional directions to *Xist* and module eigengene levels in brain (Fig. 9D). But in striking contrast*, Xist, Mecp2,* and *Kdm6a* correlated with each other and in the same direction as the placental networks (Fig. 9E). While male tissues lacked detectable *Xist* expression (grey NA), *Mecp2* and *Kdm6a* also showed opposite correlations with gene networks between male brain and placenta, suggesting this placental-brain difference was not *Xist* related. Among these three X-linked genes, *Xist* showed strong positive associations with the widespread sex-specific transcriptional responses and was directly influenced by PCB dose, suggesting a potential regulatory role in mediating sex differences in response to PCB exposures.

## Discussion

The neurotoxicity of PCBs is predicted to be related to their known effects on calcium channels and neuronal synapses [25, 26], but genome-wide analyses of the molecular changes associated with PCB exposure are rare. Our previous whole-genome analyses of DNA methylation changes resulting from a 1 mg/kg/d dose of the same PCB mixture and mouse model used in this study are consistent with the strong overlap in dysregulated gene pathways between the placenta and brain, even though the direction of change was sometimes directionally opposite [21]. In the present study, we directly examined sex and tissue differences as potential explanations for the directionally different responses of the brain and placenta. Since our study was the first to our knowledge to employ the WGCNA approach to bioinformatic analyses of PCB effects, we were able to uniquely demonstrate broad changes across multiple gene networks enriched for functions in energy and cellular metabolism that were distinctly altered in females compared with males. Our results are consistent with those of a previous transcriptome and lipidome study in the zebrafish brain that revealed that disruption of energy homeostasis could be explained by impaired pathways of mitochondrial function and lipid metabolism regulation following exposure to an environmentally-relevant mixture of PCBs and PBDEs [27]. Our results showing enriched mitochondrial and oxidative phosphorylation gene pathways are also consistent with the results of a transcriptomic analysis of newborn mouse dentate granule cells following perinatal exposure to Aroclor 1254, a commercial PCB mixture [28].

What is unique in our investigation is the integration of placental and fetal brains and the demonstration of profound sex differences in the transcriptional response to prenatal PCB exposure in both the brain and placenta. For instance, in the PCB exposed male brain, but not placenta, coexpressed gene modules were enriched for function in “Metabolism of xenobiotics by cytochrome P450” and “Glutathione metabolism” KEGG pathways, which are known responses to toxins (Fig. 5). In contrast, PCB exposed female placenta differed from female brain in modules with strong enrichments to “Oxidative phosphorylation” and “Ribosome”, suggesting that differential use of mitochondrial and translational pathways influencing energy metabolism in the female placenta are related to sex differences in how the placental-brain axis responds to PCBs.

Sex differences related to PCB exposure have been previously reported, but are poorly understood [20, 29]. Sex differences in response to prenatal PCB exposure have been mixed in human epidemiological studies examining the secondary sex ratio [30, 31]. However, a recent large population study revealed that multiple air and water pollutants are associated with changes in the sex ratio at birth, including PCBs which are associated with male bias [32]. In a recent analysis of six different prenatal chemical mixtures with metabolic syndrome (MetS) scores stratified by sex, the PCB mixture was uniquely associated with a higher MetS score in female children and a lower MetS score in male children, which is consistent with our findings of large-scale sex differences in the transcriptional modulation of metabolic genes in the placenta [29]. While differences in sex hormones are usually implicated in transcriptional sex differences, females also differ from males genetically in the presence of two X chromosomes and the lack of a Y chromosome. A prior study demonstrated that the X-linked gene *O*-GlcNAc transferase (OGT) gene is highly expressed in the female placenta and that placental OGT is required for the protective effects of prenatal stress on mitochondrial energy production in females [33]. OGT acts as a central sensor for glucose availability and cellular nutrient status, altering its activity and the entire glycosylation landscape, including pathways for complex O-glycans. Interestingly, we also observed enrichment of O-glycan biosynthesis gene pathways that may cross-talk with OGT in protective female modules responding to PCB combined with folic acid protection (Fig. 8). Notch signaling pathways were also enriched in females, whereas glycan degradation pathways that degrade Notch-related proteins were enriched in males perinatally exposed to PCBs. Together, these results are consistent with sex differences in metabolic pathway activation in the placenta-brain axis, which may be involved in the imbalance in OGT and other X- or Y-linked genes with differential expression.

To further explore an X-linked hypothesis, we tested associations of sex-specific gene modules with three X-linked genes involved in epigenetic functions: the female-specific *Xist* lncRNA involved in X chromosome inactivation, the histone demethylase encoding gene *Dnm6a* that escapes X inactivation, and the methyl CpG binding protein encoding gene *Mecp2* that is subject to X inactivation. Interestingly*, Xist* levels mirrored the nonmonotonic dose effect of PCB exposure in both brain and placenta and were positively correlated with expression in the modules showing opposite sex effects. The canonical function of *Xist* is X chromosome inactivation through the recruitment of epigenetic silencing factors to the inactive X chromosome around the time of embryo implantation [34, 35]. *Xist* was also thought to be required for maintenance of XCI dosage compensation after the initial formation of the heterochromatic inactive X chromosome. However, studies using conditional *Xist*^2flox^ transgenic mice have shown very mild transcriptional effects, including in a neuron-specific *Xist* deletion model [36]*.* In this model*, Xist* loss was observed across multiple organs, except with the addition of an exogenous chemical stressor in the gut [37]*.* These intriguing results raise the question, why is *Xist* maintained in adulthood if not for maintaining X chromosome inactivation? Remarkably, *XIST* levels are elevated in human females with psychiatric diagnoses [38] and *XIST* transcript levels are implicated in several cancer progression phenotype [39–41]*. Xist* levels are also elevated in rodent models of spinal cord injury, promoting neuronal repair but also neuroinflammation [42, 43]. A study using a dox-titratable *Xist* expression cell line model provided an elegant mechanism for how increasing *Xist* expression levels impacts transcriptional silencing effects on autosomal genes, but also a self-feedback loop on its own expression [44].These combined findings, together with the sex-differential DNA methylation PCB effects we previously observed in the placental-brain axis of this PCB exposure model [21]_,_ suggest that widespread sex differences in metabolic pathways responding to PCBs are due primarily to genetic and epigenetic differences in sex chromosome content between the sexes. Further investigations into the mechanisms involving *Xist* as a potential epigenetic modulator of the female protective effect are warranted.

In the present study, we expanded the PCB mixture doses to a total of three (0.1, 1, and 6 mg/kg/d) and discovered a strong non-monotonic dose response, with the medium dose evoking the lowest transcriptional response. Our results showing that the non-monotonic response to in utero exposure to PCBs was dependent on both sex and tissue are consistent with our prior studies of behavioral and neuronal dendritic phenotypes in the same PCB mixture mouse model [16]. Non-monotonic responses have been frequently described previously for PCBs and other endocrine-disrupting chemicals but are poorly understood. Among the six different molecular mechanisms described to explain non-monotonic effects [45], our results are most consistent with dose-dependent metabolism modulation [46] because of the sex-specificity and large number of genes and gene pathways regulating metabolism in the placental-brain axis. Because our transcriptomic analyses were performed on bulk tissues, we cannot rule out the possibility that shifts in cell populations may underlie some effects, although our enrichment analysis of genes in each of the modules suggest that few show cell type specificity (Additional file 3: Tables S9-S16). Single cell resolution of the brain and placental samples from this mouse model would be a more comprehensive way to determine if the non-monotonic effects could be due to cell-type or cell-state differences in addition to modulation of metabolism.

Our results have potential translational relevance in understanding if current levels of PCBs in pregnant women remain a concern for neurodevelopment. While PCB levels have gradually declined in the environment and human bodies over recent decades, our current finding of broadly dysregulated fetal brain transcription combined with our prior finding of social behavioral impairments in males at the lowest 0.1 mg/kg/d dose [16] suggests that PCB neurotoxicity does not necessarily decrease with lower exposure levels, because even low-level environmental exposure to PCBs can have transcriptional and behavioral effects. Interestingly, our results indicate that dietary folic acid supplementation was successful in reducing the transcriptional impacts of PCBs in fetal mice by acting on similarly disrupted metabolic pathways, including oxidative phosphorylation and O-glycan pathways. Prenatal vitamins containing folic acid are recommended for all women planning pregnancy to prevent neural tube defects and potentially other neurodevelopmental conditions like autism spectrum disorders, but only ~ 50% of women in the MARBLES cohort took prenatal vitamins in the first month of pregnancy or before which is the window of protection in humans [12]. While folic acid is supplemented in the US grain supply to prevent neural tube defects, that level does not appear to be sufficient for preventing ASD, as prenatal vitamin use was the main protective source of folic acid when including all dietary sources in the CHARGE case–control cohort [15]. While our results are limited to transcriptional analyses in a single strain of inbred mice, they are potentially supportive of public health education campaigns about the importance of taking prenatal vitamins prior to conception to prevent possible harm from low-level PCBs and other pollutants.

Our results also have implications for the early detection of neurodevelopmental disorders at birth. As the major source of nutrients and other support to the fetus, the placenta is a tissue source rich in epigenetic and transcriptional biomarkers that can be indicative of the in utero environment. Since the placenta is a readily available tissue source discarded at birth, its biomarkers can be used to assess neurodevelopmental disorder risk via diagnostic screening approaches at birth. Since age of ASD diagnoses is quite variable, screening placental samples for biomarkers could potentially lead to earlier diagnoses and interventions for ASD and other neurodevelopmental disorders. Tissue-consensus WGCNA can identify exposure-associated modules that show cross-tissue concordance between brain and placenta, which are enriched for shared pathways that may capture meaningful signature of in utero stress in response to neurotoxins like PCBs. The presence of exposure-associated modules shared by brain and placenta suggests that hub genes could potentially serve as predictive biomarkers in placental tissue as a minimally invasive indicator of in utero molecular risk to the developing brain.

Our findings are also expected to have clinical relevance beyond neurodevelopmental disorders. Enriched KEGG pathways for PCB-altered genes revealed the neurodegenerative disorders Parkinson and Huntington disorders, as well as coronavirus and prion disease, all of which may involve oxidative phosphorylation and metabolic pathways in their pathogenesis. A prior study of the effects of PCB-180 on human iPSC-derived neurons also identified Parkinson disease pathway genes [47]. Since sex differences in the prevalence and/or severity of both neurodevelopmental and neurodegenerative disorders are apparent, our results highlight the importance of the placental-brain axis for understanding sex via environmental interactions in brain health.

## Conclusion

This study was designed to understand the molecular pathways in the brain-placenta axis that may explain the male bias in NDD risk, utilizing an existing mouse model of prenatal exposure to a human-relevant mixture of PCBs. Transcriptomic analyses of embryonic brain and matched placenta were performed via systems biology-based approaches, resulting in several novel findings. First, prenatal PCB exposure resulted in widespread dysregulation of correlated gene networks enriched in energy and cellular metabolism, but the direction of these effects varied between males and females, as well as between the brain and placenta. Second, the female-specific placental transcriptional responses were more protective against PCB and were uniquely enriched in citrate metabolism, oxidative phosphorylation, and decreased energy consumption corresponded to relatively muted transcriptional responses in the female compared with the male brain. Third, there was a strong non-monotonic effect of PCB dosage, with the lowest and highest dosages showing the strongest transcriptional responses, but this effect was both sex-dependent and correlated with *Xist* levels in brain and placenta. Finally, folic acid supplementation protected against most of the gene network transcriptional changes, but these interaction effects were most prominent in the female placenta in both the DEG interaction tests and the WGCNA. Together, these results provide evidence for a protective effect of the female placenta-brain axis in modulating the expression of metabolic pathways in a way that reduces the transcriptional impact on the developing brain.

## Methods

All animal work was approved by the UC Davis IACUC protocol #20584.

### Mouse exposure model and tissue harvest

The PCB MARBLES mixture, consisting of PCB 28 (48.2%), PCB 11 (24.3%), PCB 118 (4.9%), PCB 101 (4.5%), PCB 52 (4.5%), PCB 153 (3.1%), PCB 180 (2.8%), PCB 149 (2.1%), PCB 138 (1.7%), PCB 84 (1.5%), PCB 135 (1.3%) and PCB 95 (1.2%), was formulated to mimic the 12 most abundant congeners identified from the serum of pregnant women in the ASD-enriched MARBLES cohort, as previously described [48]. C57BL/6 J female mice (Jackson Laboratory) aged 6 to 8 weeks were orally exposed to 4 different doses (0, 0.1, 1.0, or 6.0 mg/kg/d) of the PCB mixture in peanut oil (Spectrum Organic) and peanut butter (Trader Joe’s Organic Creamy). The levels of each PCB congener in mouse brain, serum, and liver have been previously quantitated in adult females exposed to these doses [49] and represent the range of exposure levels measured in human brain and serum [8, 21, 50, 51]. The PCB mixture was stored as a concentrated stock in peanut oil and then diluted in peanut butter to make dosing mixes for the four dose groups (0, 0.1, 1, and 6 mg/kg/d). The dosing mixtures were prepared for the entire experiment, stored in the refrigerator and remixed daily. For alterations to folic acid in the chow, the mice were fed ad libitum, with the AIN-93G purified diet, which contained either the standard 2 mg/kg (control, Standard FA) or the 6 mg/kg (FA-enriched) total folic acid (ENVIGO 190757 and 94045, respectively). The human equivalence of these daily FA levels is 400 µg/day for the Standard FA versus 1200 µ g/day for the FA-enriched, which was modelled to represent the protective effect associated with ≥ 600 µg/day total dietary FA, with higher doses associated with greater significance^15.^ Females were dosed daily for at least 2 weeks before breeding commenced. Daily dosing then continued through breeding and gestation, until embryonic day 18 (E18). On E18, females were euthanized in a CO_2_ euthanasia chamber, but not all were pregnant, limiting the number of dams per dosing group (Table 5). Immediately after euthanasia of pregnant dams, the embryos were removed and the whole brain and placenta were dissected and flash frozen. The embryo and tissue weights were recorded, as were the relative positions within the uterine horns. Table 5 shows the total number of samples harvested for transcriptomic analyses per treatment group. DNA was isolated from embryonic tail and genotyped for sex using PCR primers to *Sry*.

**Table 5:** Number of samples harvested from 2-4 dams per group, sexes combined

Dosing Group	Dams Per Group	96 Matched Brain/Placenta Samples	Female Samples	Male Samples
0 PCB/Standard FA	3	13 Brain 13 Placenta	6	7
0.1 PCB/Standard FA	2	15 Brain 15 Placenta	10	5
1 PCB/Standard FA	3	15 Brain 15 Placenta	9	6
6 PCB/Standard FA	2	15 Brain 15 Placenta	6	9
0 PCB/FA-enriched	4	18 Brain 18 Placenta	10	8
0.1 PCB/FA-enriched	4	20 Brain 20 Placenta	10	10

### RNA isolation and RNA-sequencing

RNA was isolated from snap frozen placenta and brain tissue by homogenizing tissue via a TissueLyser II (Qiagen) followed by the All-Prep DNA/RNA/miRNA Universal Kit (Qiagen). The isolated RNA was quantified and purity measured via the Bioanalyzer Eukaryotic Total RNA Nano Assay (Agilent). Libraries were constructed via the KAPA mRNA HyperPrep Kit (Roche) and NEXTFLEX Unique Dual Index Barcodes (PerkinElmer). Libraries were pooled and sequenced on a NovaSeq 6000 S4 flow cell (Illumina) for 150 bp paired end reads resulting in approximately 25 million uniquely mapped reads per sample.

### Bioinformatic Analyses

Raw RNA-seq fastq files were processed by trimming adapters via Trim Galore (v0.6.5) and alignment to the mouse genome (mm10) followed by gene count quantification via STAR (v2.7.3a) [52]. QC metrics were generated with MultiQC (v1.9).

#### Differential gene expression analysis

Differential expression and subsequent pathway enrichment analyses were carried out via R version 4.1.0. Counts were filtered and normalized via limma voom followed by the fitting of a linear mixed model via dream weights (variancePartition package) to model litter as a random effect. The eBayes() function was used to test for differential expression, and a cutoff false discovery rate (FDR < 0.05) was used to define DEGs. Three different models were fit: 1) gene expression ~ group with contrasts fit to compare each group, 2) gene expression ~ PCB dose as a continuous variable, and 3) gene expression ~ PCB + Folic Acid + PCB*Folic Acid to examine genes that had a significant effect modification of Folic Acid on PCB dose. ComplexUpset was used to create UpSet plots of gene overlaps across groups. enrichR followed by the GO term slimming with rrvgo was used for pathway enrichment analysis.

#### Weighted gene correlation network analysis

Weighted gene correlation networks [22] were generated for several subsets of the RNA-seq data to compare similar modules of highly correlated genes across either sex or tissue and to test the interaction effects of a single PCB dose (0.1 mg/kg/) and folic acid (FA) supplementation. Four separate consensus networks were generated for PCB dosage effects between the sexes (PCB sex-consensus networks for placenta and brain) and PCB dosage effects between the tissues (PCB tissue-consensus networks for males and females). Four separate consensus networks were generated for PCB + FA supplementation effects (FA-enriched/Standard FA, vehicle PCB mg/kg/day with FA-enriched/Standard FA 0.1 ppm PCB) between the sexes and between the tissues (PCB + FA sex-consensus networks for placenta and brain) and (PCB + FA tissue-consensus networks for males and females). Normalized gene expression matrices comprising 13,241 genes were used in all the consensus networks. Hierarchical clustering of samples was performed to identify potential sample outliers for each consensus network.

Scale-free topology was used to determine the soft-power threshold to which pairwise correlations between genes are raised. A scale-free topology model fit of at least 0.85 was used to determine the soft-power threshold (Additional file 2: Fig. S4-S11). Signed networks were used to maintain the direction of co-expression information among genes. Tukey biweight midcorrelation, a robust method that downweights potential outliers, was used for the network construction. Modules were identified using unsupervised clustering and the number of modules was not explicitly specified. Modules were identified using unsupervised clustering and the number of modules was not explicitly specified. A range of minimum number of genes in a module was evaluated to assess clustering resolution and an intermediate resolution was selected based on module stability and biological interpretability (Table 6). Module eigengenes, essentially the first principal component of a module that serves as a reduced representation of the gene expression of all genes within the module, were computed for each module. Spearman correlation, which can capture monotonic but potentially nonlinear module–trait relationships, was then used to find correlations between module eigengenes (MEs) and treatment group comparisons (traits). Hub genes for each module were defined as the gene having the highest absolute module eigengene based connectivity (kME) within that module.

**Table 6:** Parameters for module generation in WGCNA

**Co-expression Network**	**Samples** **(N =)**	**Power**	**Minimum Genes in Module**	**Number of Modules Generated**
PCB Sex-Consensus Brain	27 Male 31 Female	22	50	24
PCB Sex-Consensus Placenta	27 Male 31 Female	18	150	24
PCB Tissue-Consensus Male	27 Brain 27 Placenta	16	100	22
PCB Tissue-Consensus Female	31 Brain 31 Placenta	20	100	19
FA Sex-Consensus Brain	28 Male 35 Female	28	50	29
FA Sex-Consensus Placenta	30 Male 36 Female	28	150	18
FA Tissue-Consensus Male	30 Brain 30 Placenta	28	100	12
FA Tissue-Consensus Female	35 Brain 36 Placenta	24	100	13

entrezIDs were converted to gene symbols via the biomart database in R to allow gene ontology analysis and KEGG pathway analysis to be performed for every module within each consensus network. Modules that had correlations in the same direction (between sex or tissue) in at least 5 columns in the heatmap were considered to act similarly (between sex or tissue), and those that had correlations in opposite directions (between sex or tissue) in at least 5 columns were considered to act differently (between sex or tissue). The top 3 KEGG terms enriched in these modules, along with their p-values and log odds ratios are shown (an FDR adjusted p-value of 0.05 was considered statistically significant).

## Supplementary Information

Additional file 1. RNA-seq quality control reports for each sample.

Additional file 2: Supplementary Figures S1-S12. Fig. S1. Sample-to-Sample Spearman Correlation Heatmap of gene expression, annotated by tissue, sex, PCB dose, litter, and uterine horn position. Fig. S2. Analysis of variance by experimental variable an PCA analysis of litter effects. Fig. S3. Examples of select PCB dose responsive DEGs. Fig. S4. PCB sex-consensus networks for brain. Fig. S5. PCB sex-consensus network for placenta. Fig. S6. PCB tissue-consensus networks for males. Fig. S7. PCB tissue-consensus networks for females. Fig. S8. Folic Acid sex-consensus network for brains. Fig. S9. Folic Acid sex-consensus networks for placenta. Fig. S10. Folic Acid tissue-consensus networks for males. Fig. S11. Folic Acid tissue-consensus networks for females. Fig. S12. Additional pairwise comparisons for sex-consensus folic acid networks. This is an expanded version of Fig. 6 but with all the pairwise comparisons. Fig. S13. Additional pairwise comparisons for tissue-consensus folic acid networks. This is an expanded version of Fig. 7 but with all the pairwise comparisons.

Additional file 3: Supplementary Table S1-S16.

## Data Availability

All RNAseq data have been deposited in GEO accession GSE315769 [53].The code used to generate the analyses in this study is available on GitHub: (https://github.com/kellychau/RNAseqWeightedGeneCorrelationNetworksPCBBrainPlacenta) [54] and is released under the MIT license. A DOI‑minted archival copy is available at (https://doi.org/10.5281/zenodo.19011412) [55].
