# Supplementary material for: Sex and tissue resolved co-expression networks reveal a female placental–brain axis protective against prenatal PCB exposure
Source: Genome Biol. 2026 Apr 7;27:171. doi: 10.1186/s13059-026-04052-8 (PMC13188613; doi:10.1186/s13059-026-04052-8)
Supplement: Supplementary file 1 — Additional file 1. RNA-seq quality control reports for each sample. [file 13059_2026_4052_MOESM1_ESM.pdf]

MultiQC

RNA-seq

(http://mmi-lab.ucdavis.edu/wordpress/)

(http://multiqc.info)

RNA-seq

QC for RNA-seq workflow

Multiple QC reports to summarise trimming and alignment.

> This is the LaSalle lab version.

Workflow Developer
Ben Laufer
E-mail
blaufer@ucdavis.edu
Application Type
RNA-seq
Project Type
PEBBLES FA Dosing Study
Sequencing Platform
NovaSeq 6000
Sequencing Setup
PE 150 DI
Library Kit
KAPA mRNA HyperPrep
Genome
mm10

Report generated on 2021-06-17, 07:58 based on data in: `/share/lasallelab/Kari/PEBBLES_FA_Dosing_RNA-seq`

General Statistics

Showing 960 samples.

Hover over a data point for more information

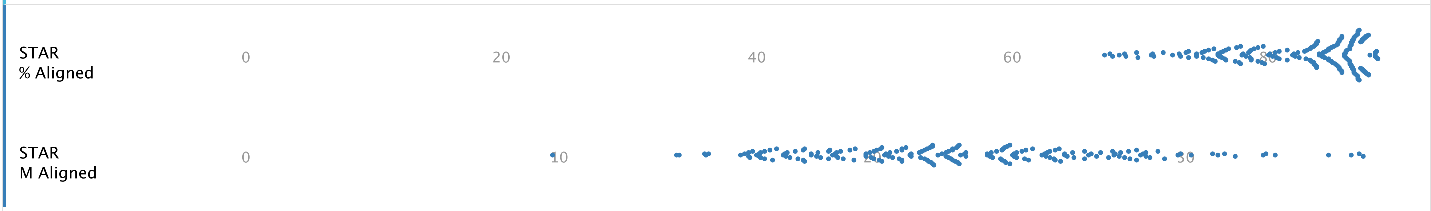

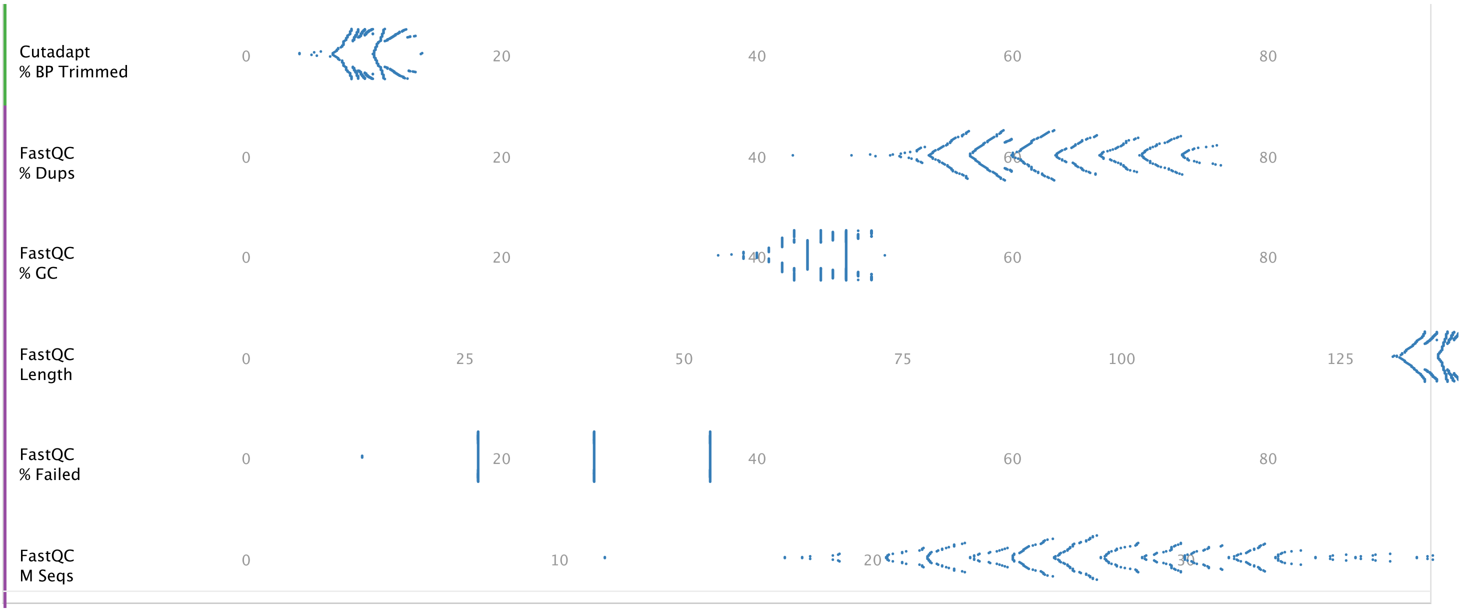

STAR

STAR (https://github.com/alexdobin/STAR) is an ultrafast universal RNA-seq aligner.

Alignment Scores

Flat image plot. Toolbox functions such as highlighting / hiding samples will not work (see the docs (http://multiqc.info/docs/#flat--interactive-plots)).

Number of Reads | Percentages

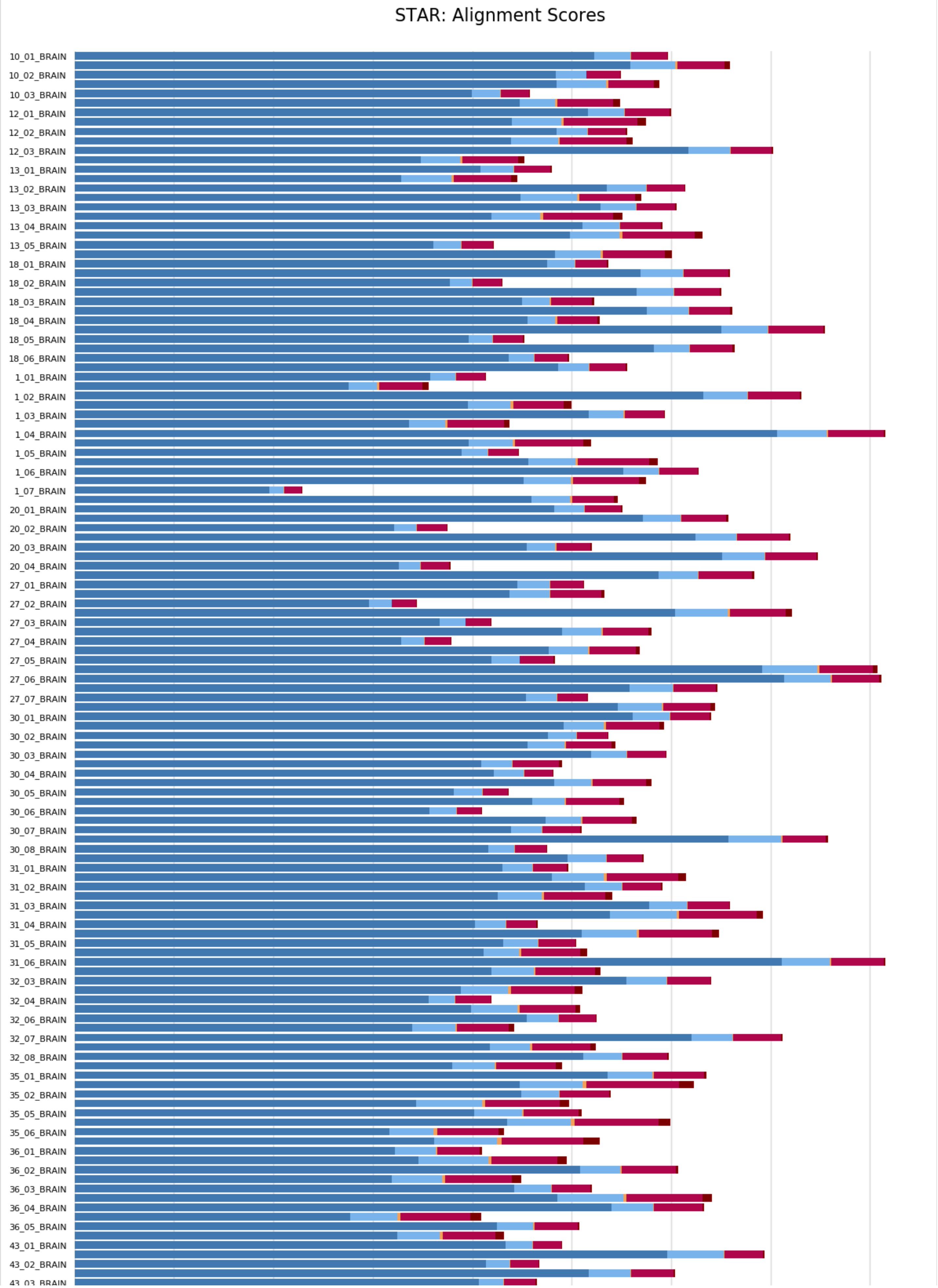
STAR: Alignment Scores
10_01_BRAIN
10_02_BRAIN
10_03_BRAIN
12_01_BRAIN
12_02_BRAIN
12_03_BRAIN
13_01_BRAIN
13_02_BRAIN
13_03_BRAIN
13_04_BRAIN
13_05_BRAIN
18_01_BRAIN
18_02_BRAIN
18_03_BRAIN
18_04_BRAIN
18_05_BRAIN
18_06_BRAIN
1_01_BRAIN
1_02_BRAIN
1_03_BRAIN
1_04_BRAIN
1_05_BRAIN
1_06_BRAIN
1_07_BRAIN
20_01_BRAIN
20_02_BRAIN
20_03_BRAIN
20_04_BRAIN
27_01_BRAIN
27_02_BRAIN
27_03_BRAIN
27_04_BRAIN
27_05_BRAIN
27_06_BRAIN
27_07_BRAIN
30_01_BRAIN
30_02_BRAIN
30_03_BRAIN
30_04_BRAIN
30_05_BRAIN
30_06_BRAIN
30_07_BRAIN
30_08_BRAIN
31_01_BRAIN
31_02_BRAIN
31_03_BRAIN
31_04_BRAIN
31_05_BRAIN
31_06_BRAIN
32_03_BRAIN
32_04_BRAIN
32_06_BRAIN
32_07_BRAIN
32_08_BRAIN
35_01_BRAIN
35_02_BRAIN
35_05_BRAIN
35_06_BRAIN
36_01_BRAIN
36_02_BRAIN
36_03_BRAIN
36_04_BRAIN
36_05_BRAIN
43_01_BRAIN
43_02_BRAIN
43_03_BRAIN

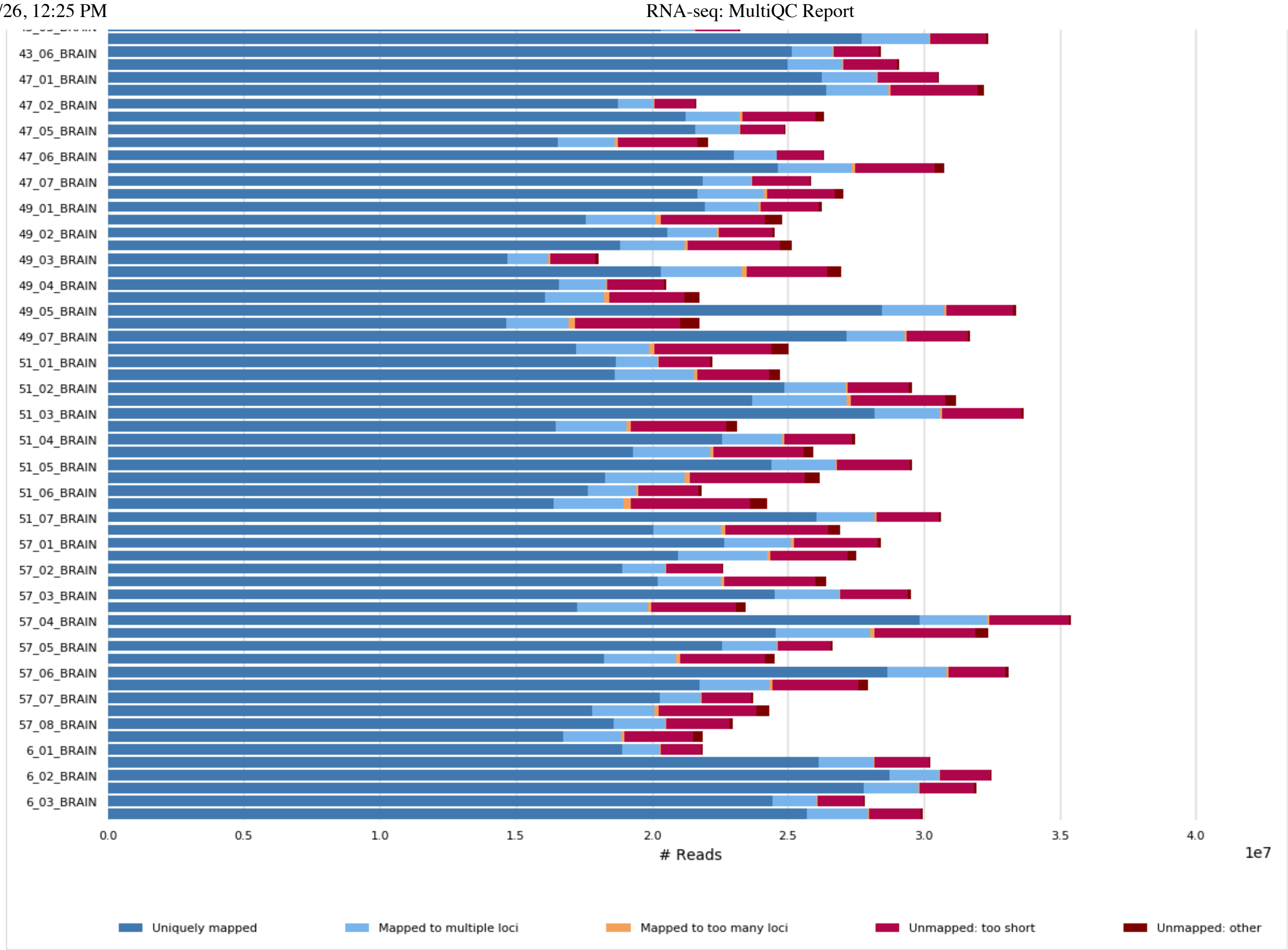

Gene Counts

Statistics from results generated using `--quantMode GeneCounts` . The three tabs show counts for unstranded RNA-seq, counts for the 1st read strand aligned with RNA and counts for the 2nd read strand aligned with RNA.

Flat image plot. Toolbox functions such as highlighting / hiding samples will not work (see the docs (http://multiqc.info/docs/#flat--interactive-plots)).

Number of Reads | Percentages

Unstranded | Same Stranded | Reverse Stranded

STAR: Gene Counts

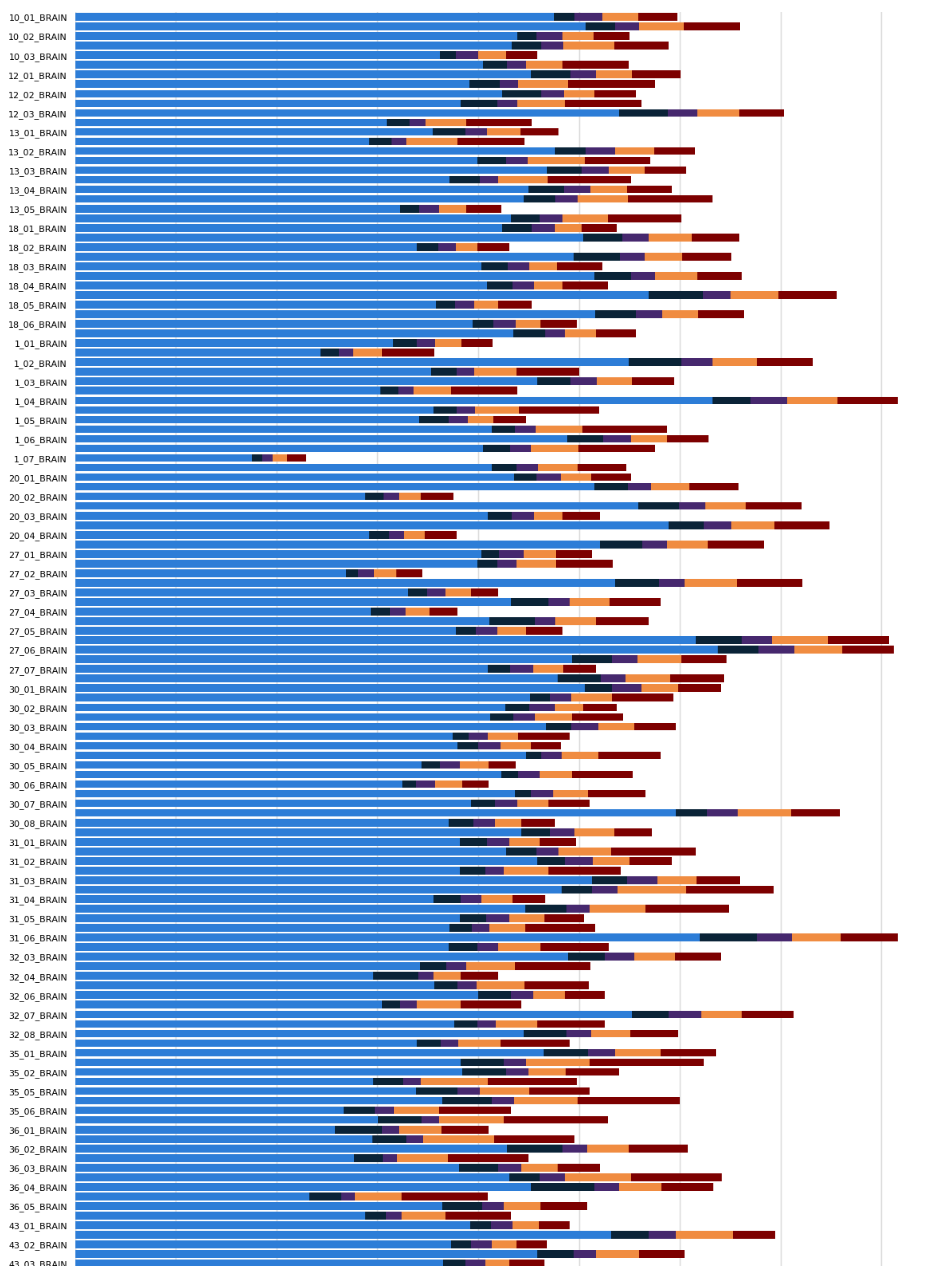

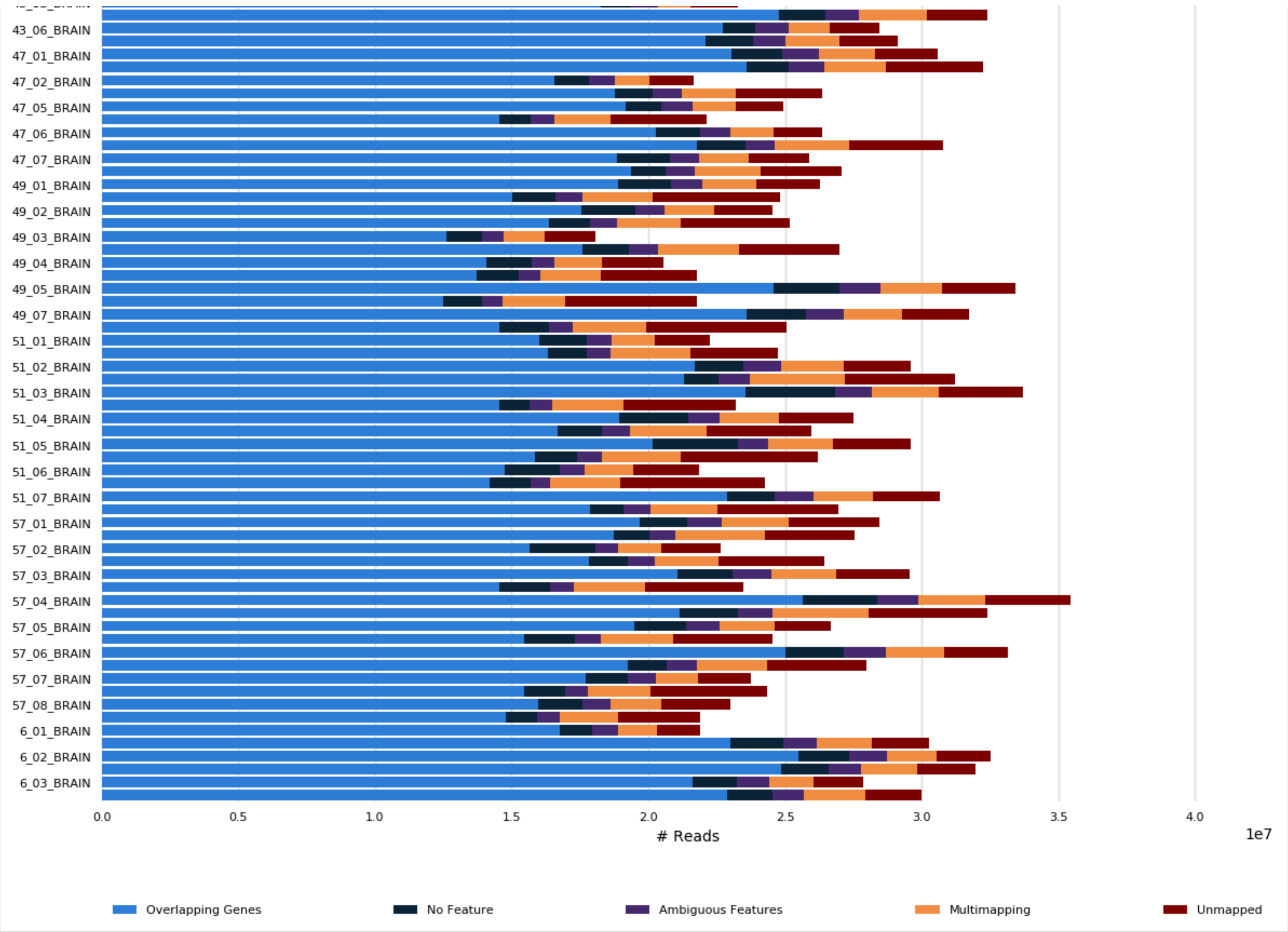

Cutadapt

Cutadapt (https://cutadapt.readthedocs.io/) is a tool to find and remove adapter sequences, primers, poly-Atails and other types of unwanted sequence from your high-throughput sequencing reads.

Filtered Reads

This plot shows the number of reads (SE) / pairs (PE) removed by Cutadapt.

Flat image plot. Toolbox functions such as highlighting / hiding samples will not work (see the docs (http://multiqc.info/docs/#flat--interactive-plots)).

Counts | Percentages

Cutadapt: Filtered Reads

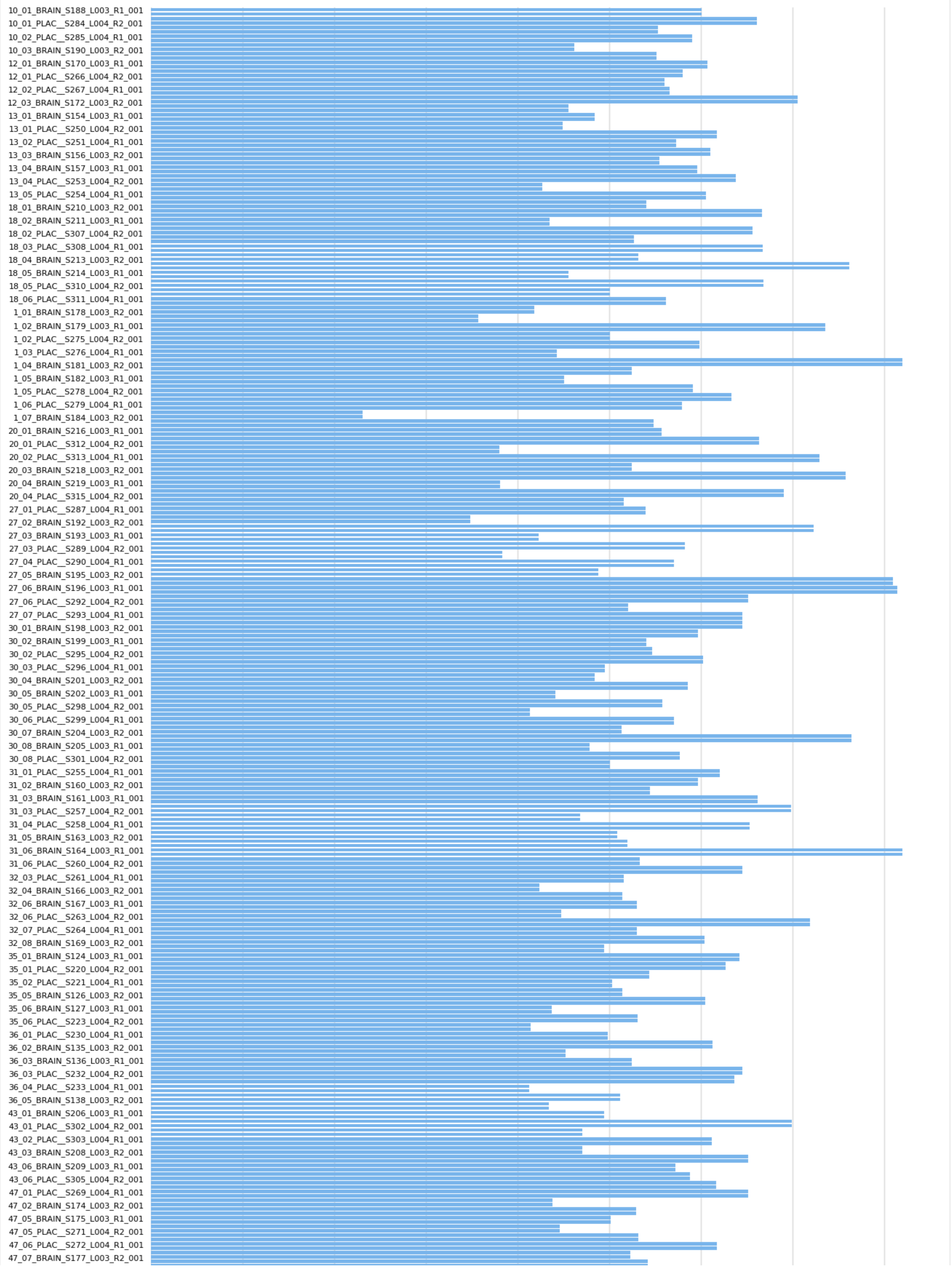

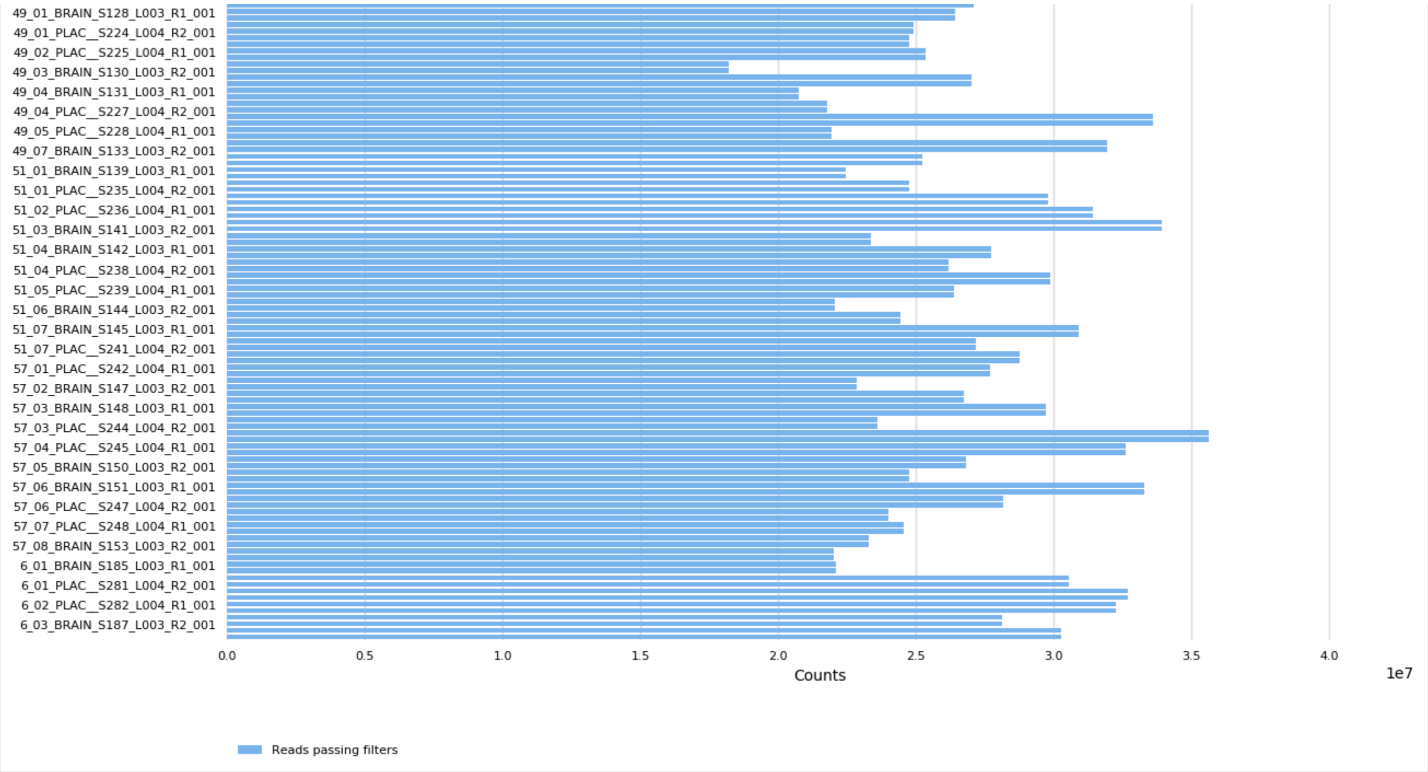

Trimmed Sequence Lengths

Help

This plot shows the number of reads with certain lengths of adapter trimmed.

Flat image plot. Toolbox functions such as highlighting / hiding samples will not work (see the docs (http://multiqc.info/docs/#flat--interactive-plots)).

Counts | Obs/Exp

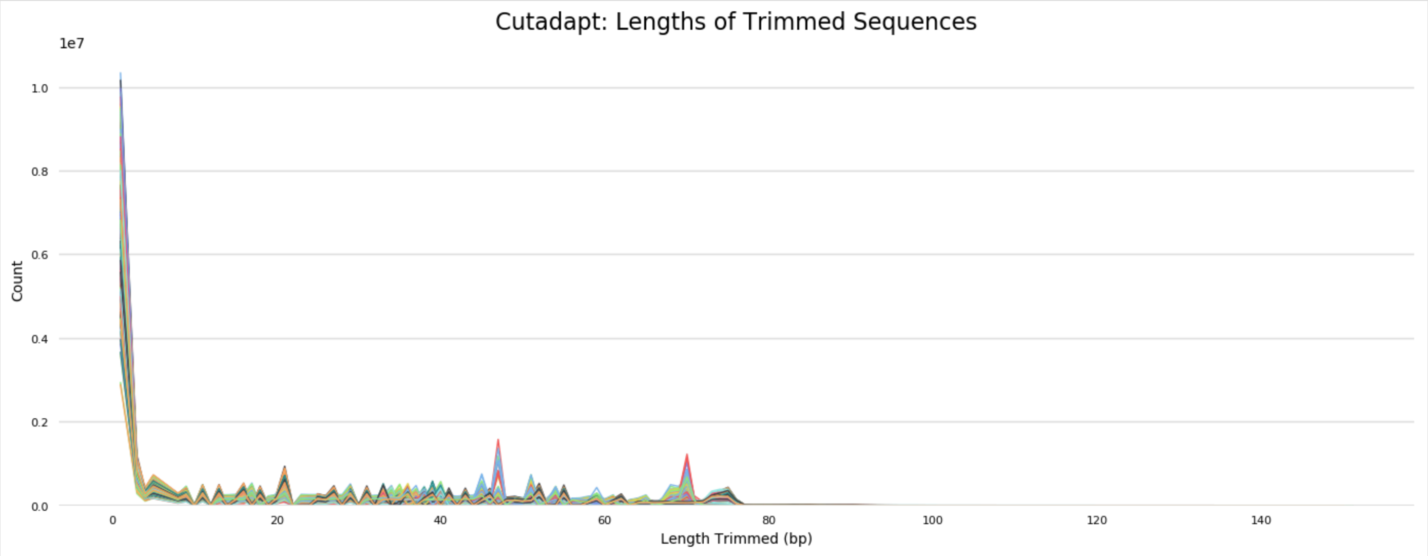

FastQC

FastQC (http://www.bioinformatics.babraham.ac.uk/projects/fastqc/) is a quality control tool for high throughput sequence data, written by Simon Andrews at the Babraham Institute in Cambridge.

Sequence Counts

Help

Sequence counts for each sample. Duplicate read counts are an estimate only.

Flat image plot. Toolbox functions such as highlighting / hiding samples will not work (see the docs (http://multiqc.info/docs/#flat--interactive-plots)).

Number of reads | Percentages

FastQC: Sequence Counts

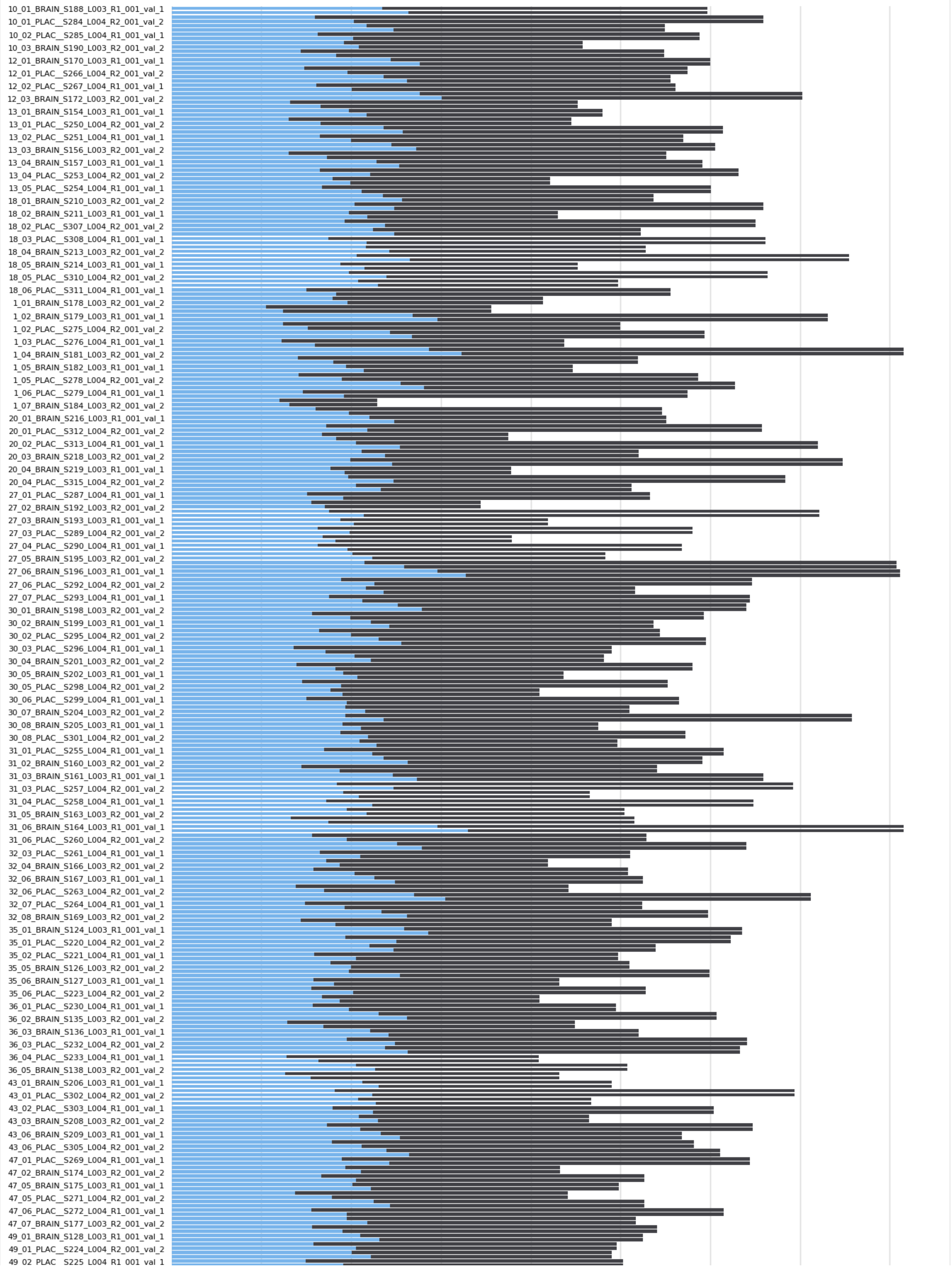

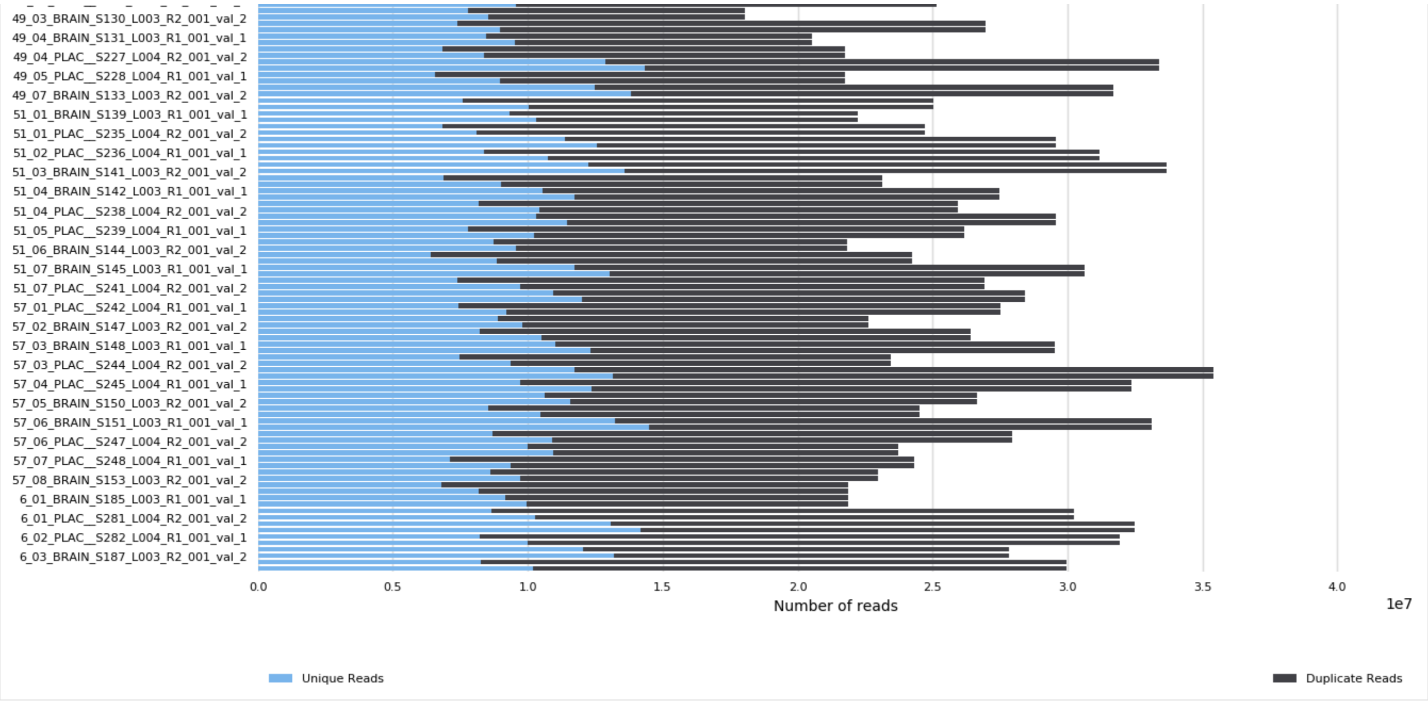

Sequence Quality Histograms

384

Help

The mean quality value across each base position in the read.

Flat image plot. Toolbox functions such as highlighting / hiding samples will not work (see the docs (http://multiqc.info/docs/#flat--interactive-plots)).

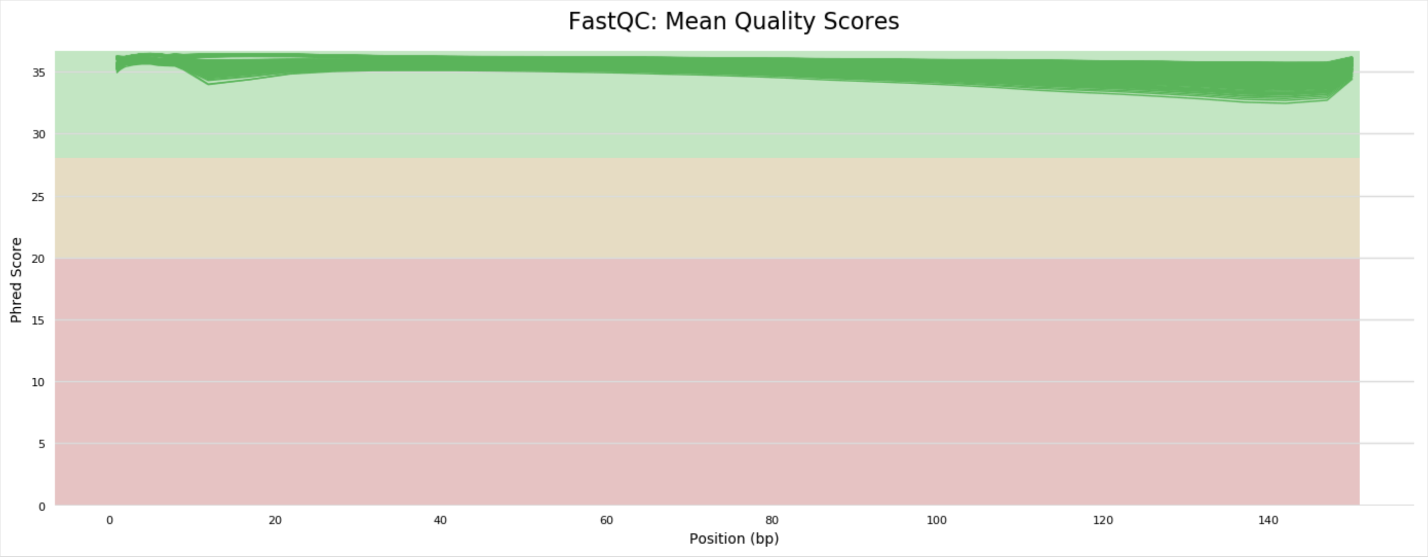

Per Sequence Quality Scores

384

Help

The number of reads with average quality scores. Shows if a subset of reads has poor quality.

Flat image plot. Toolbox functions such as highlighting / hiding samples will not work (see the docs (http://multiqc.info/docs/#flat--interactive-plots)).

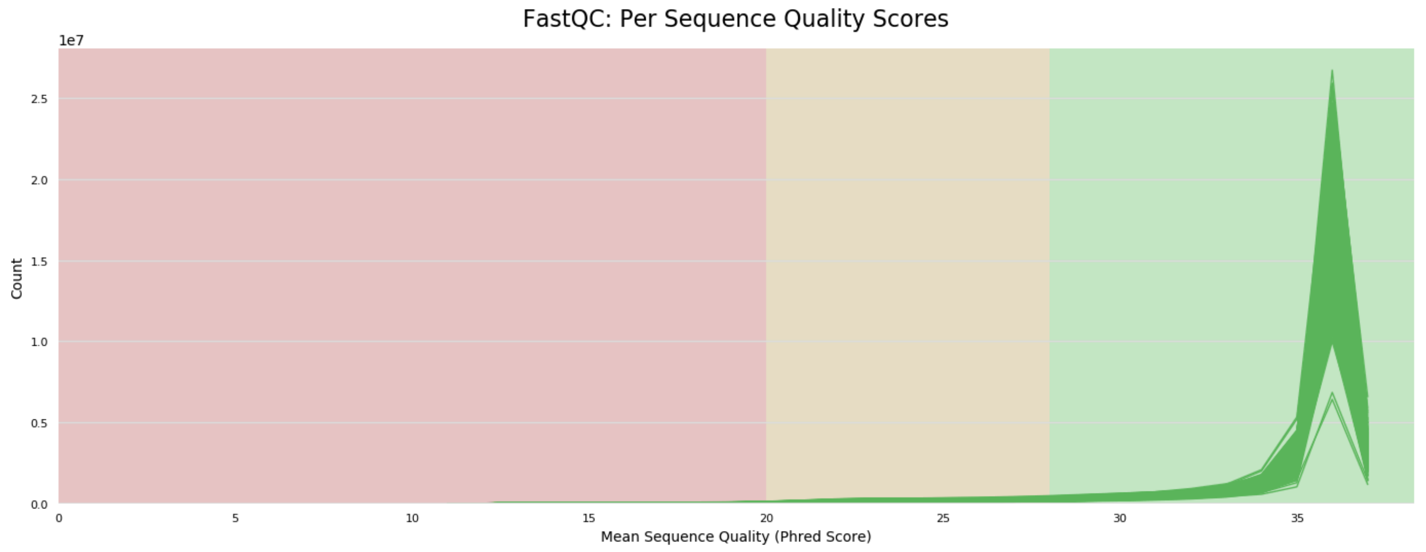

Per Base Sequence Content 0 384

Help

The proportion of each base position for which each of the four normal DNA bases has been called.

Click a sample row to see a line plot for that dataset.

Rollover for sample name

Position: - %T: - %C: - %A: - %G: -

Export Plot

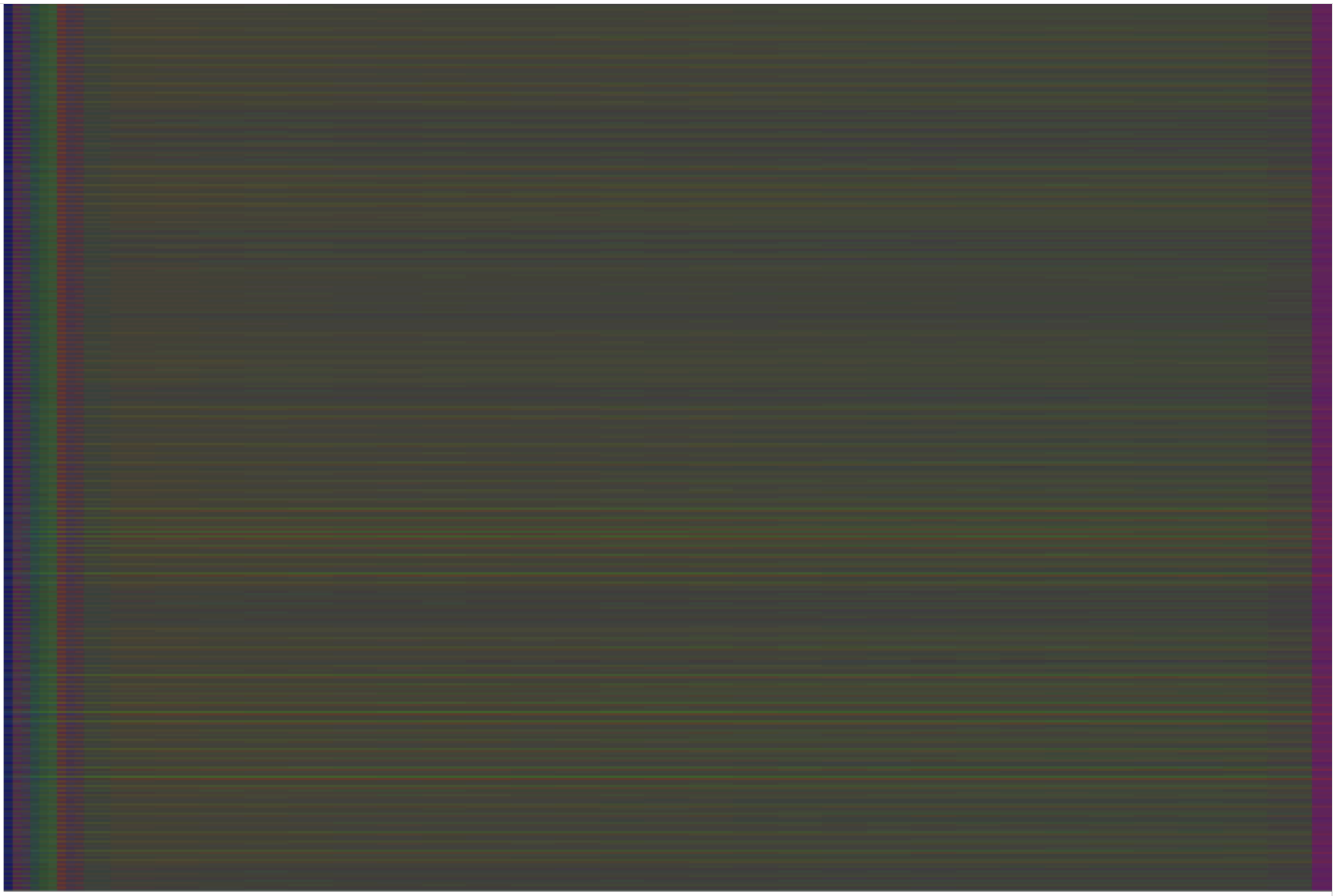

Per Sequence GC Content 17142 225

Help

The average GC content of reads. Normal random library typically have a roughly normal distribution of GC content.

Flat image plot. Toolbox functions such as highlighting / hiding samples will not work (see the docs (http://multiqc.info/docs/#flat--interactive-plots)).

Percentages Counts

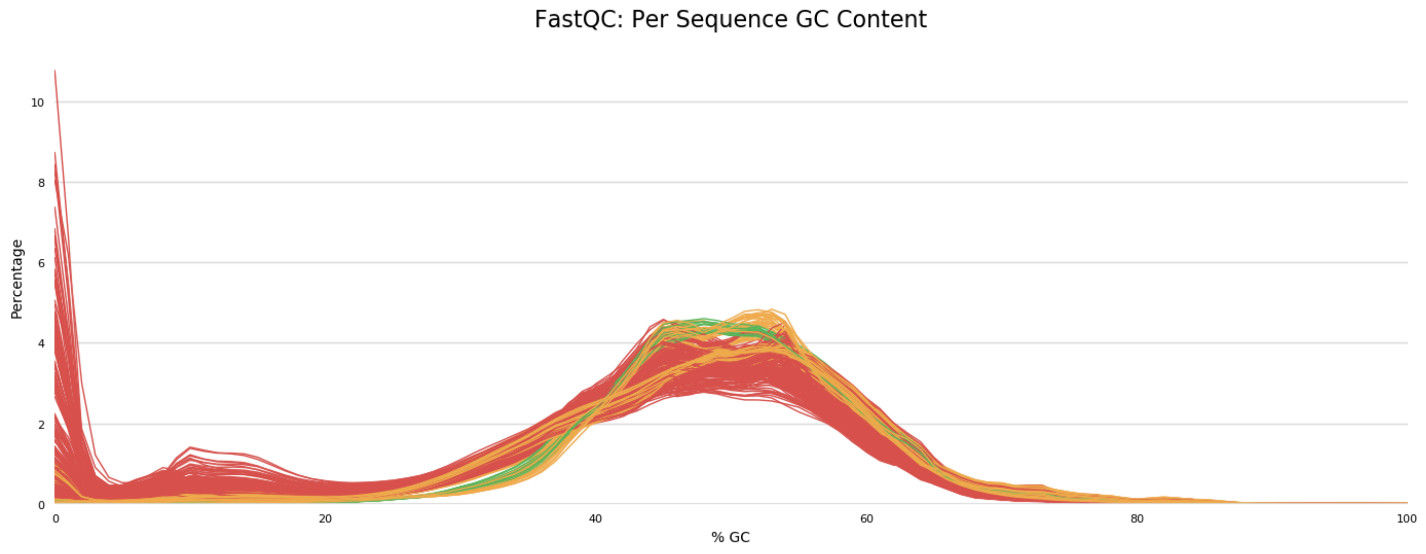

Per Base N Content 384

Help

The percentage of base calls at each position for which an `N` was called.

Flat image plot. Toolbox functions such as highlighting / hiding samples will not work (see the docs (http://multiqc.info/docs/#flat--interactive-plots)).

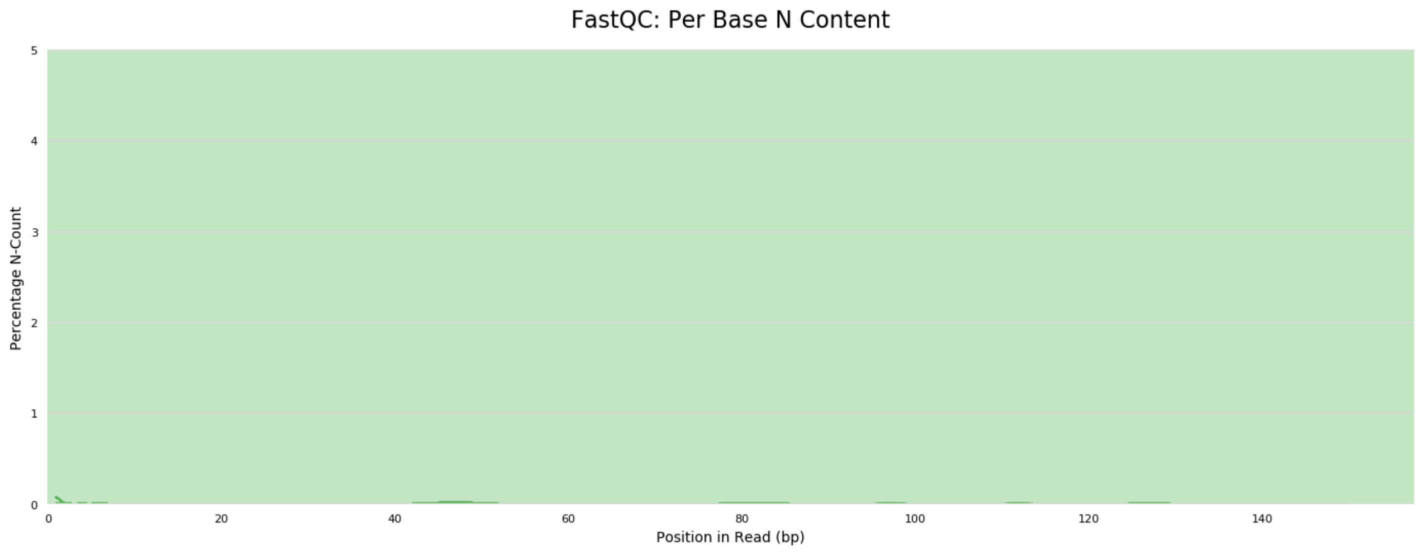

Sequence Length Distribution 0 384

The distribution of fragment sizes (read lengths) found. See the FastQC help (http://www.bioinformatics.babraham.ac.uk/projects/fastqc/Help/3%20Analysis%20Modules/7%20Sequence%20Length%20Distribution.html)

Flat image plot. Toolbox functions such as highlighting / hiding samples will not work (see the docs (http://multiqc.info/docs/#flat--interactive-plots)).

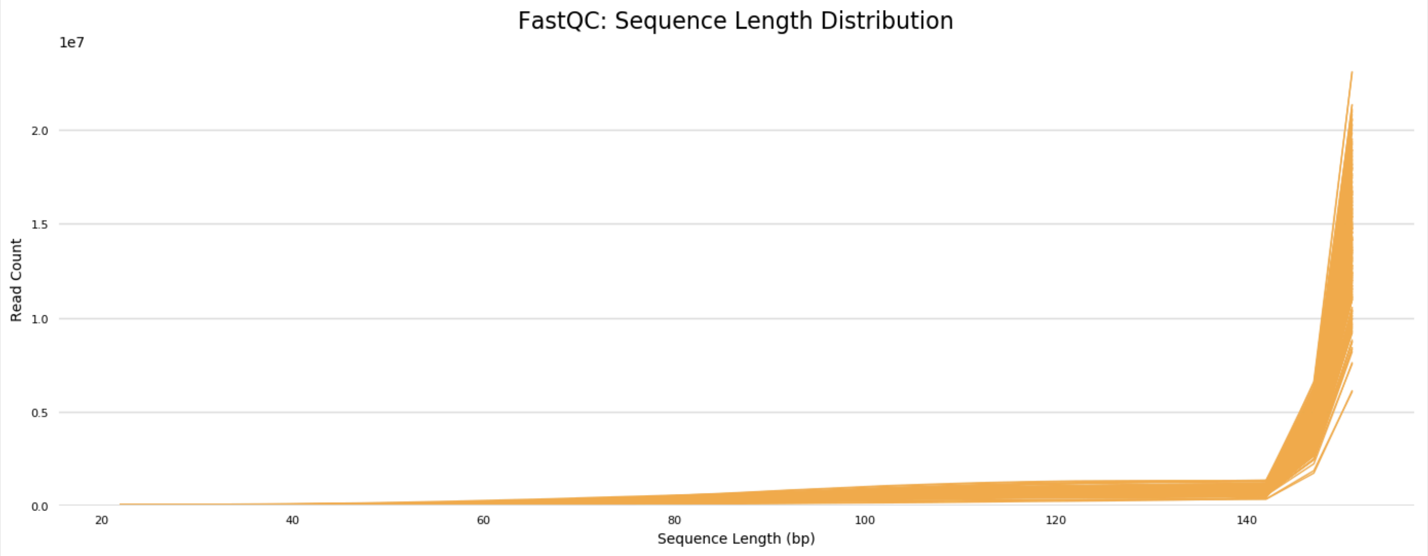

Sequence Duplication Levels 380

Help

The relative level of duplication found for every sequence.

Flat image plot. Toolbox functions such as highlighting / hiding samples will not work (see the docs (http://multiqc.info/docs/#flat--interactive-plots)).

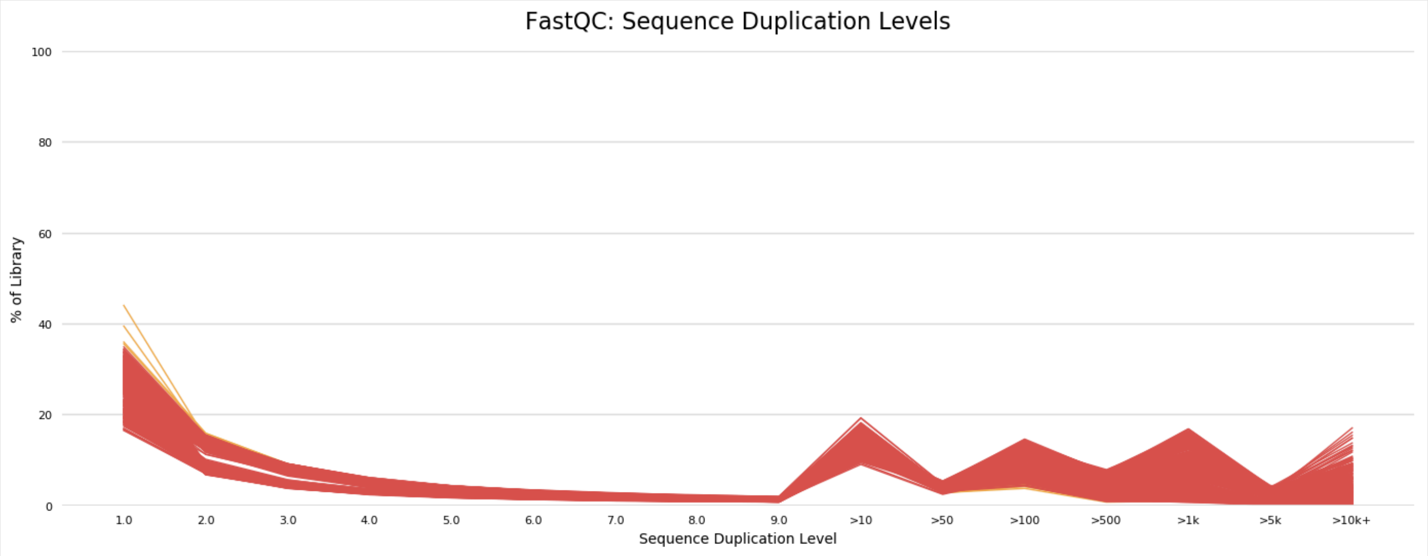

Overrepresented sequences 79 202 103

Help

The total amount of overrepresented sequences found in each library.

Flat image plot. Toolbox functions such as highlighting / hiding samples will not work (see the docs (http://multiqc.info/docs/#flat--interactive-plots)).

FastQC: Overrepresented sequences

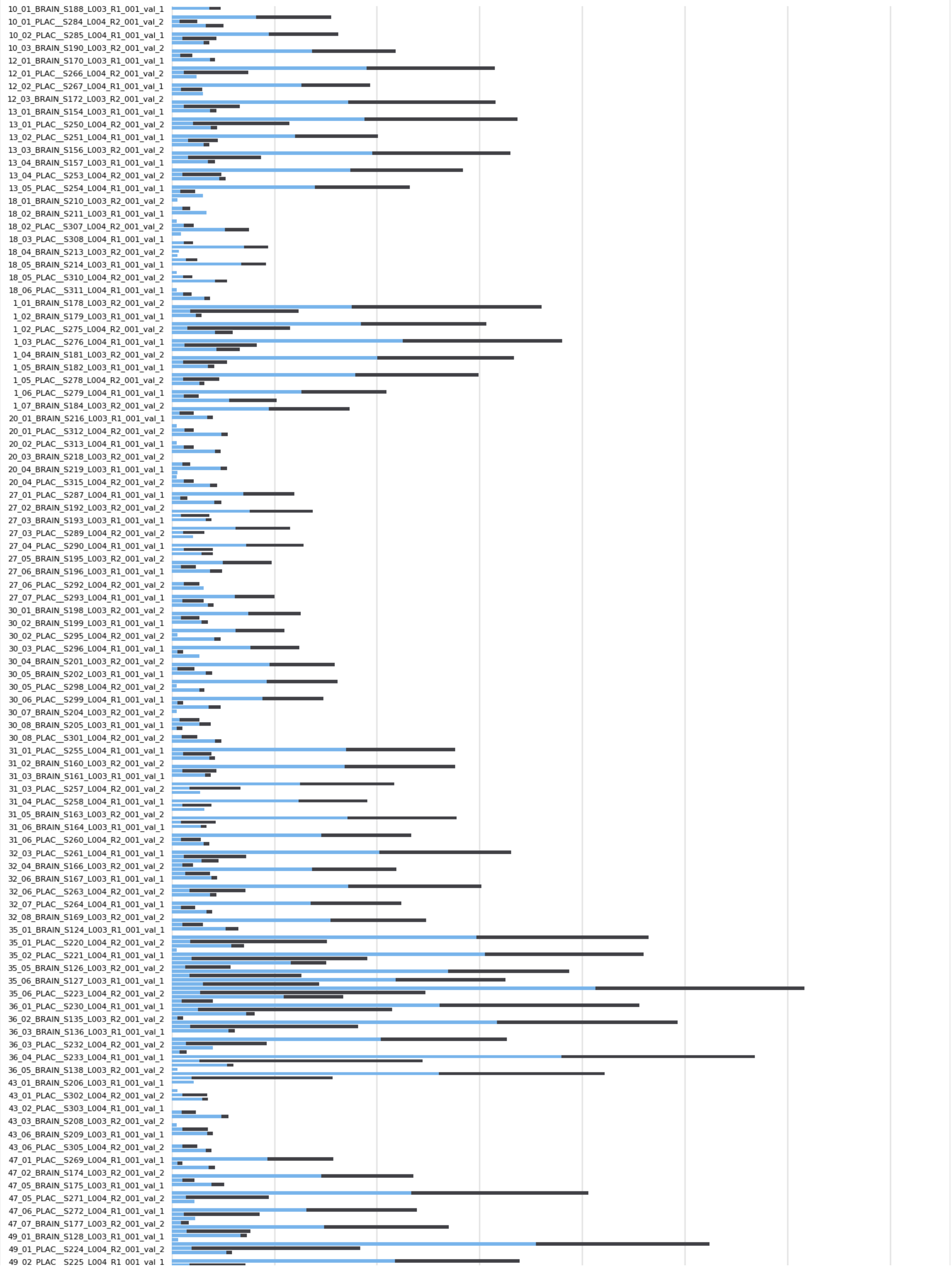

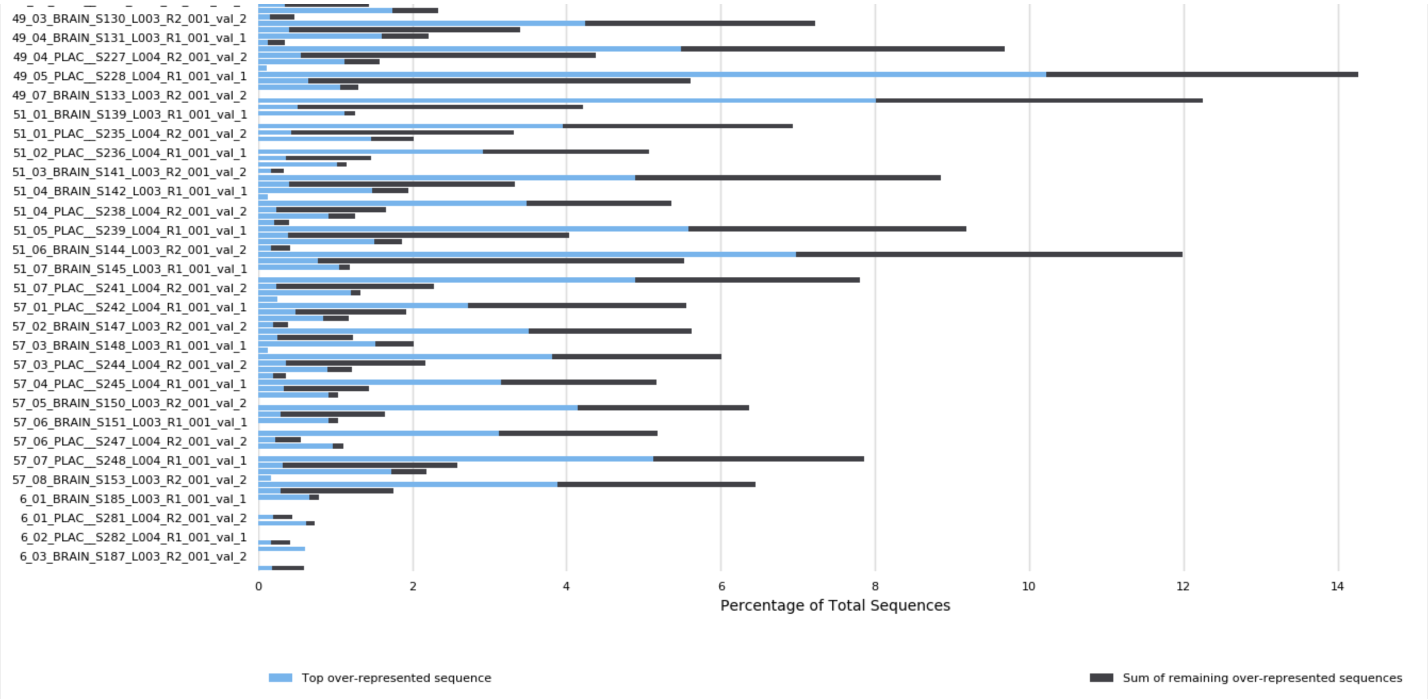

Adapter Content

384

❓ Help

The cumulative percentage count of the proportion of your library which has seen each of the adapter sequences at each position.

No samples found with any adapter contamination > 0.1%

Status Checks

❓ Help

Status for each FastQC section showing whether results seem entirely normal (green), slightly abnormal (orange) or very unusual (red).

Sort by highlight

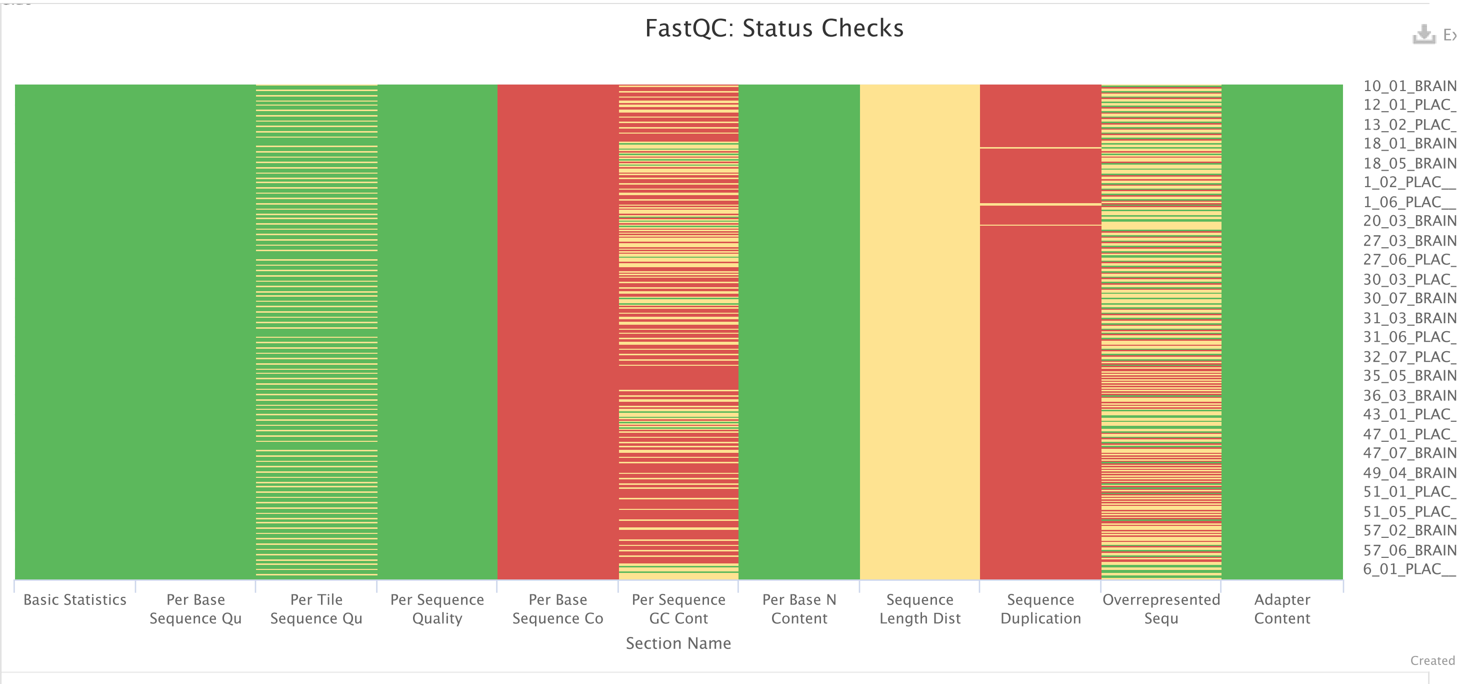

MultiQC v1.9 (http://multiqc.info) - Written by Phil Ewels (http://phil.ewels.co.uk), available on GitHub (https://github.com/ewels/MultiQC).

This report uses HighCharts (http://www.highcharts.com/), jQuery (https://jquery.com/), jQuery UI (https://jqueryui.com/), Bootstrap (http://getbootstrap.com/), FileSaver.js (https://github.com/eligrey/FileSaver.js) and clipboard.js (https://clipboardjs.com/).

SciLifeLab (http://www.scilifelab.se/)
