# Supplementary material for: Sex and tissue resolved co-expression networks reveal a female placental–brain axis protective against prenatal PCB exposure
Source: Genome Biol. 2026 Apr 7;27:171. doi: 10.1186/s13059-026-04052-8 (PMC13188613; doi:10.1186/s13059-026-04052-8)
Supplement: Supplementary file 2 — Additional file 2: Supplementary Figures S1-S12. Fig. S1. Sample-to-Sample Spearman Correlation Heatmap of gene expression, annotated by tissue, sex, PCB dose, litter, and uterine horn position. Fig. S2. Analysis of variance by experimental variable an PCA analysis of litter effects. Fig. S3. Examples of select PCB dose responsive DEGs. Fig. S4. PCB sex-consensus networks for brain. Fig. S5. PCB sex-consensus network for placenta. Fig. S6. PCB tissue-consensus networks for males. Fig. S7. PCB tissue-consensus networks for females. Fig. S8. Folic Acid sex-consensus network for brains. Fig. S9. Folic Acid sex-consensus networks for placenta. Fig. S10. Folic Acid tissue-consensus networks for males. Fig. S11. Folic Acid tissue-consensus networks for females. Fig. S12. Additional pairwise comparisons for sex-consensus folic acid networks. This is an expanded version of Fig. 6 but with all the pairwise comparisons. Fig. S13. Additional pairwise comparisons for tissue-consensus folic acid networks. This is an expanded version of Fig. 7 but with all the pairwise comparisons. [file 13059_2026_4052_MOESM2_ESM.pdf]

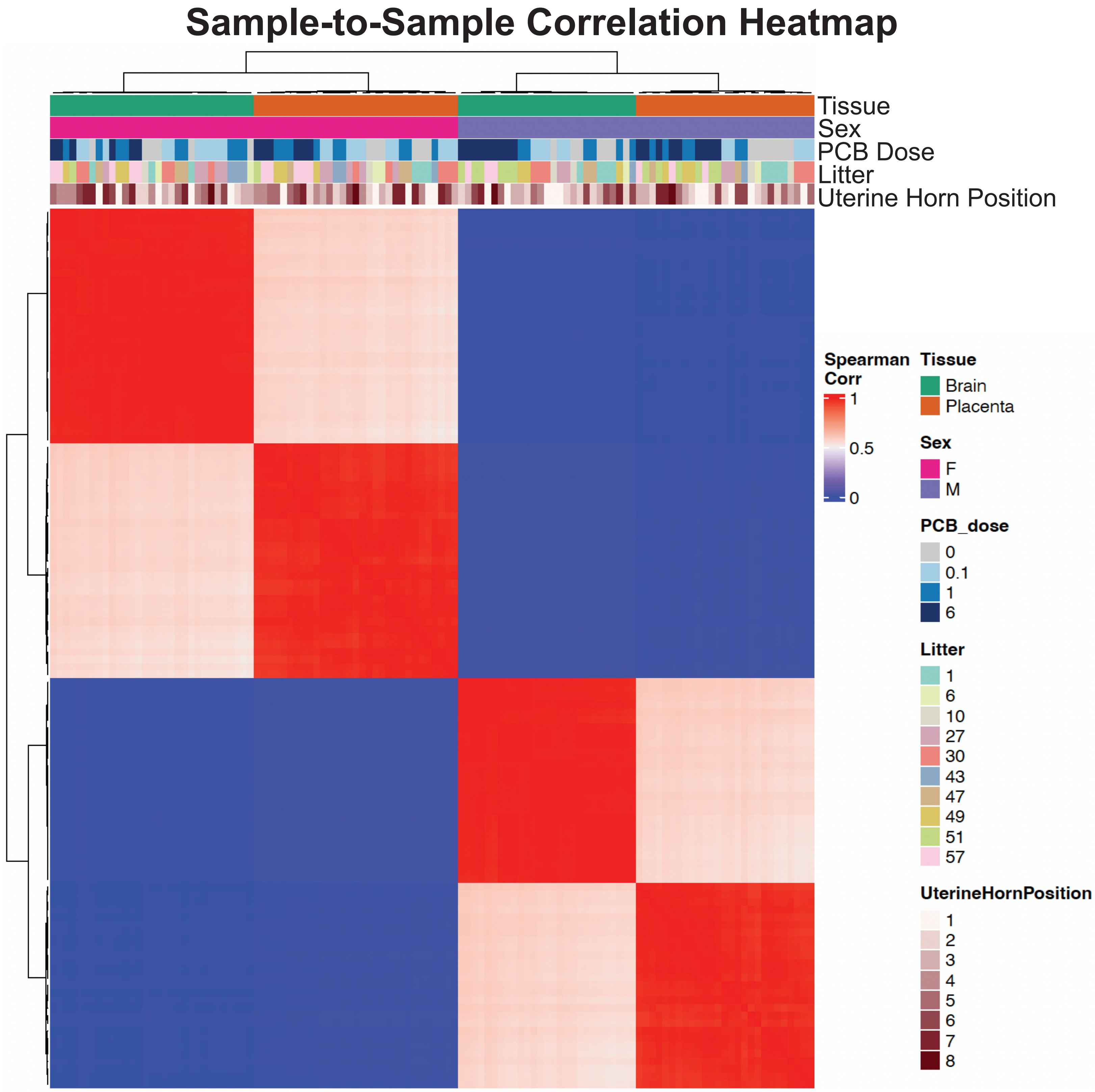

Fig. S1: Sample-to-Sample Spearman Correlation Heatmap of gene expression, annotated by tissue, sex, PCB dose, litter, and uterine horn position.

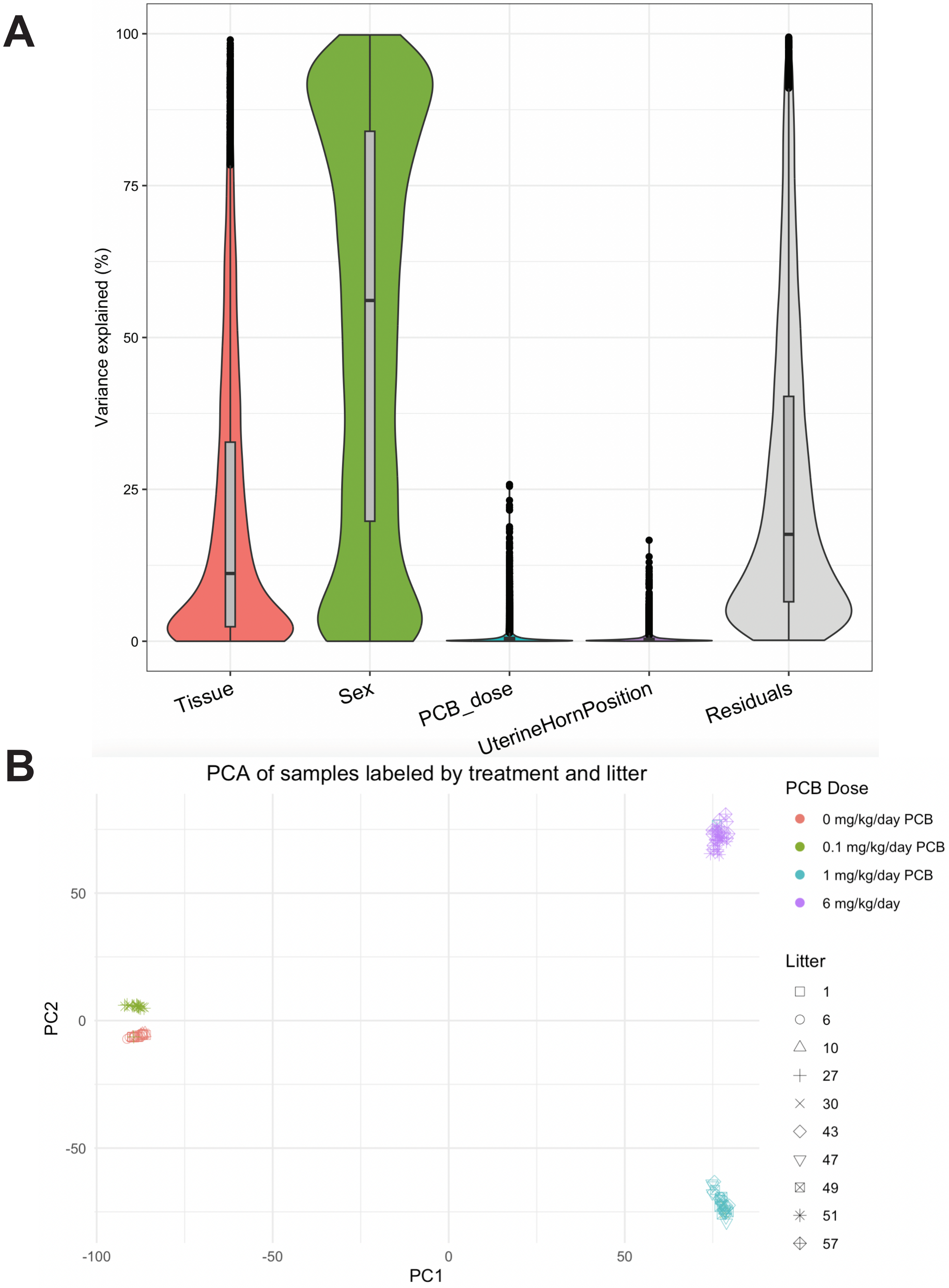

Fig. S2: A) Variance partitioning of gene expression showing the fraction of variance attributable to tissue, sex, PCB dose, and uterine horn position across genes. **B)** Principal component analysis of gene expression data shows samples clustering by PCB dose (color), while litter (shape) is distributed within each dose group.

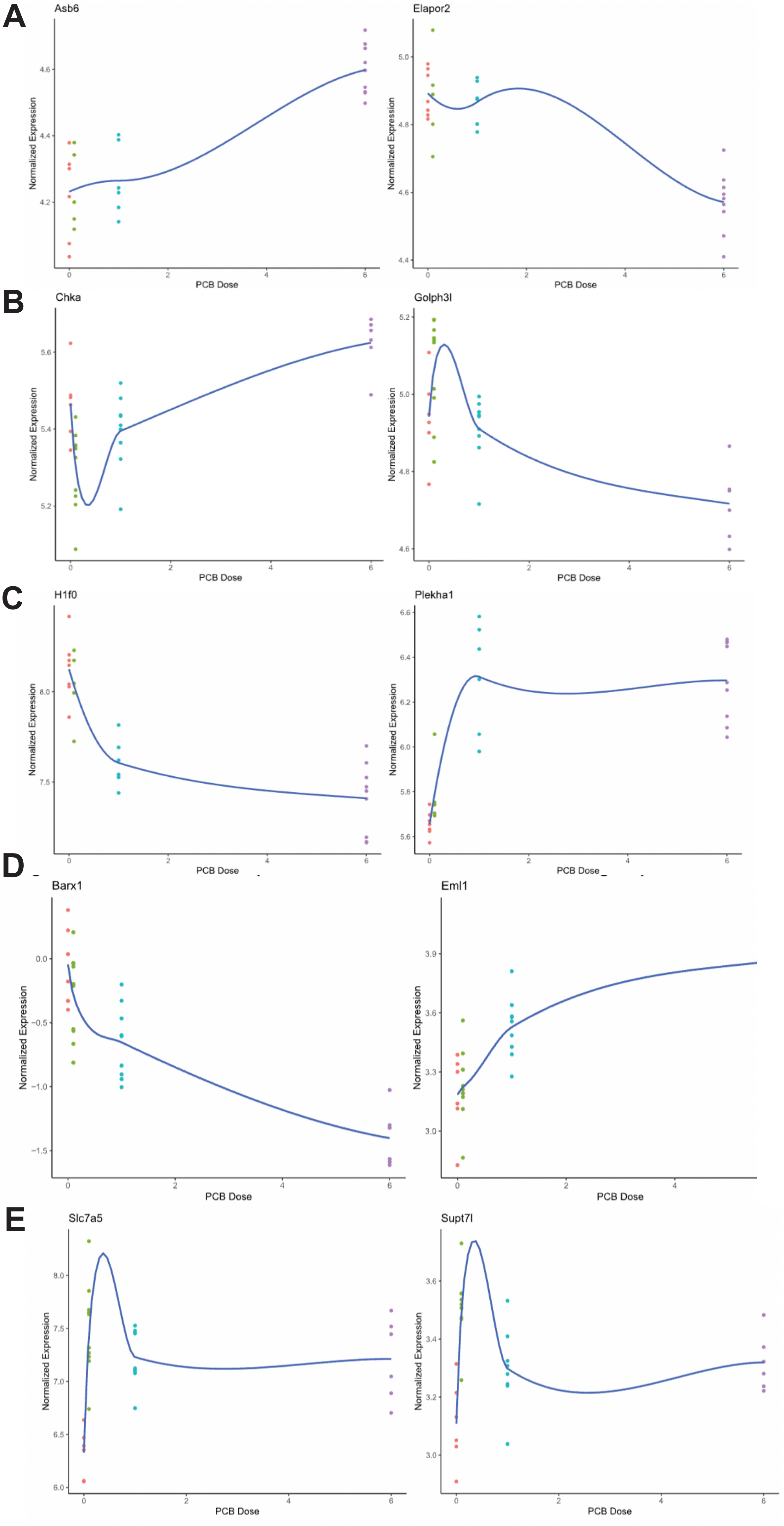

Fig. S3: A) Normalized expression levels of select male brain DEGs. **B)** Normalized expression levels of select female brain DEGs. **C)** Normalized expression levels of select male placenta DEGs. **D&E)** Normalized expression levels of select female placenta DEGs. In A-E, dots represent observations from individual embryos and lines represent loess best-fit curves. (N=5-9 embryos/group/sex)

A.

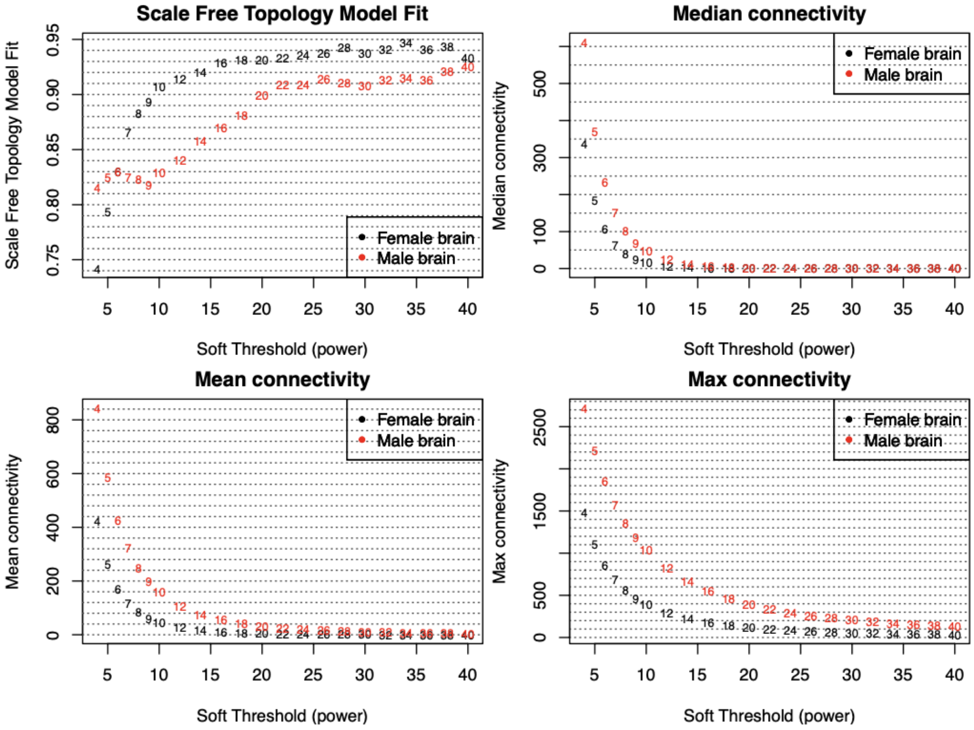

B.

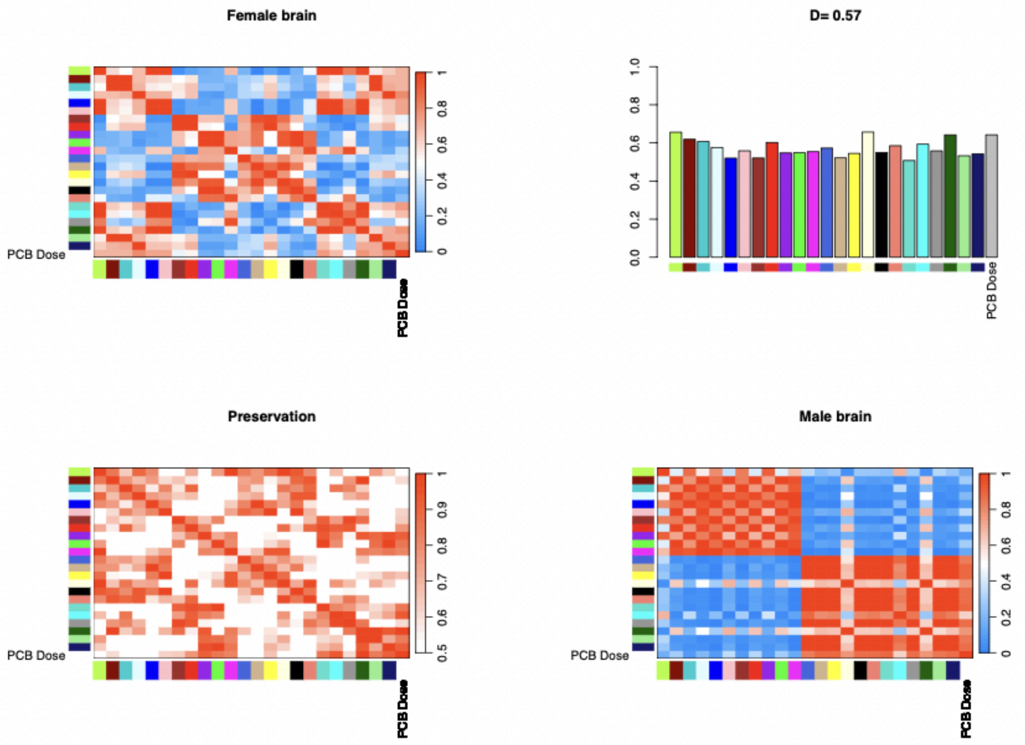

Fig. S4: PCB sex-consensus networks for brain. A) Network topology analysis. A power of 22 was selected to construct scale-free networks. **B)** Module preservation statistics. The overall preservation of the eigengene networks is denoted by D=0.57.

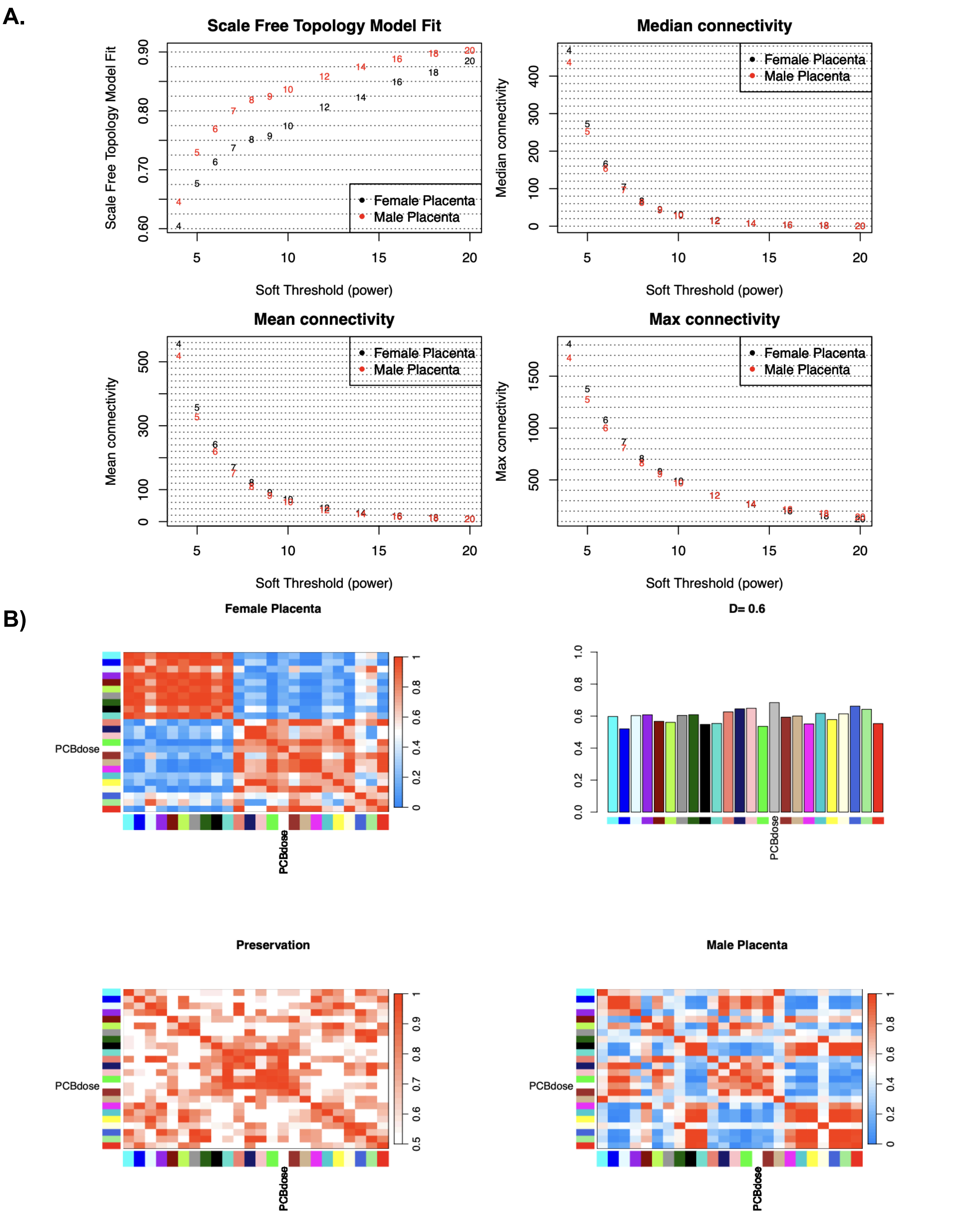

Fig. S5: PCB sex-consensus network for placenta. A) Network topology analysis. A soft-thresholding power of 18 was selected to construct scale-free networks. **B)** Module preservation statistics. The overall preservation of the eigengene networks is denoted by D=0.6.

A.

B.

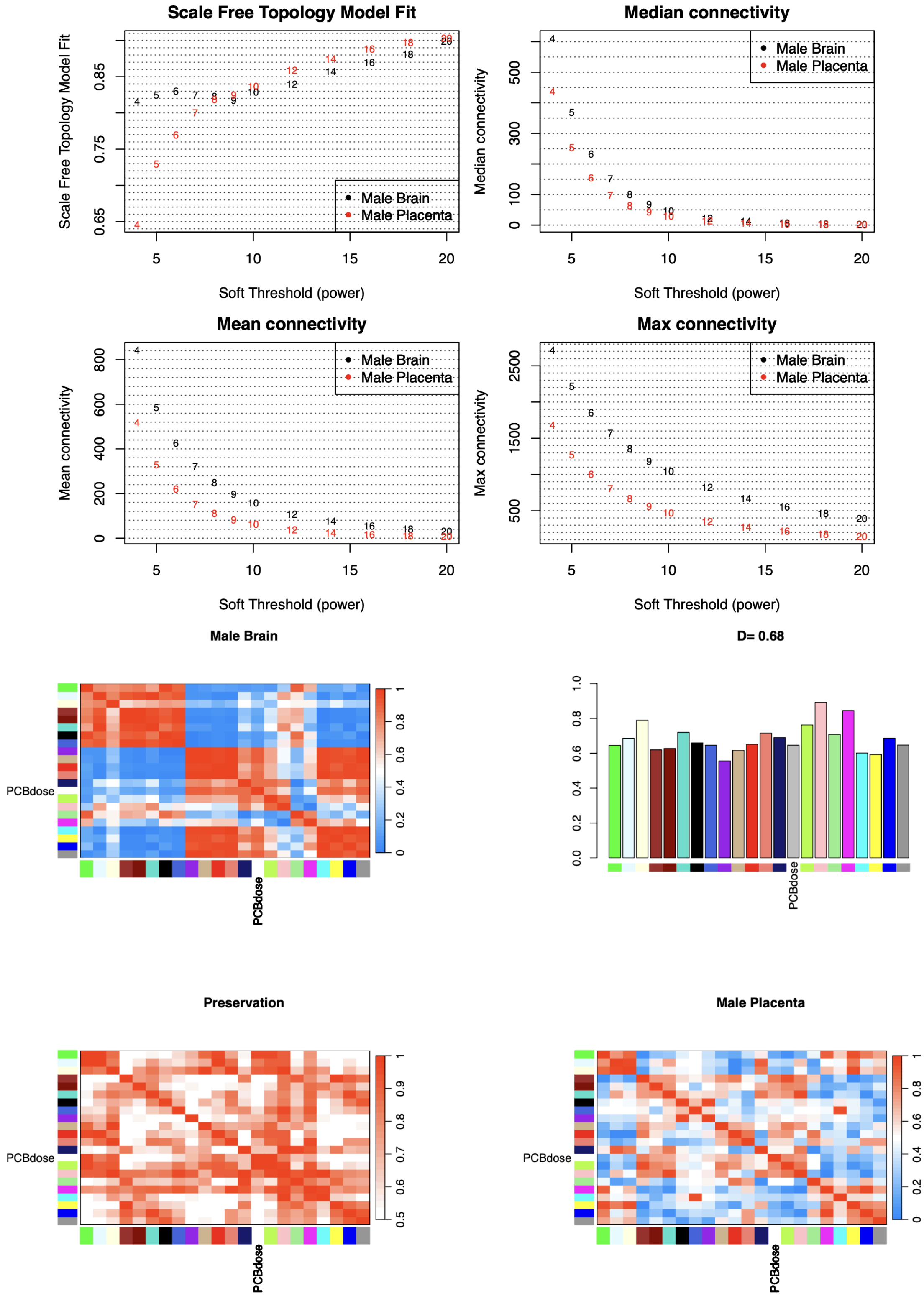

Fig. S6: PCB tissue-consensus networks for males. A) Network topology analysis. A power of 16 was selected to construct scale-free networks. **B)** Module preservation statistics. The overall preservation of the eigengene networks is denoted by D=0.68.

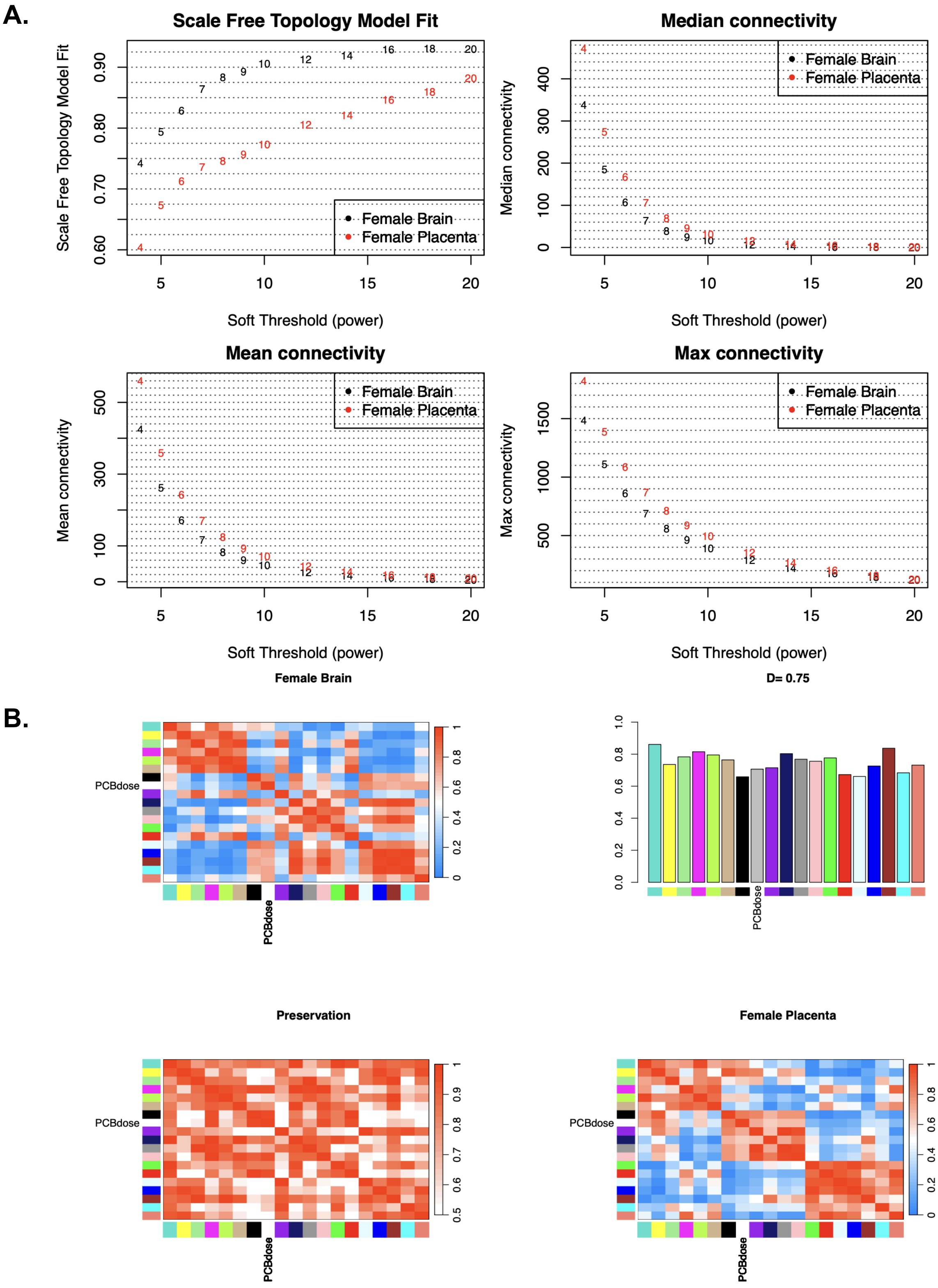

Fig. S7: PCB tissue-consensus networks for females. A) Network topology analysis. A power of 20 was selected to construct scale-free networks. **B)** Module preservation statistics. The overall preservation of the eigengene networks is denoted by D=0.75.

A.

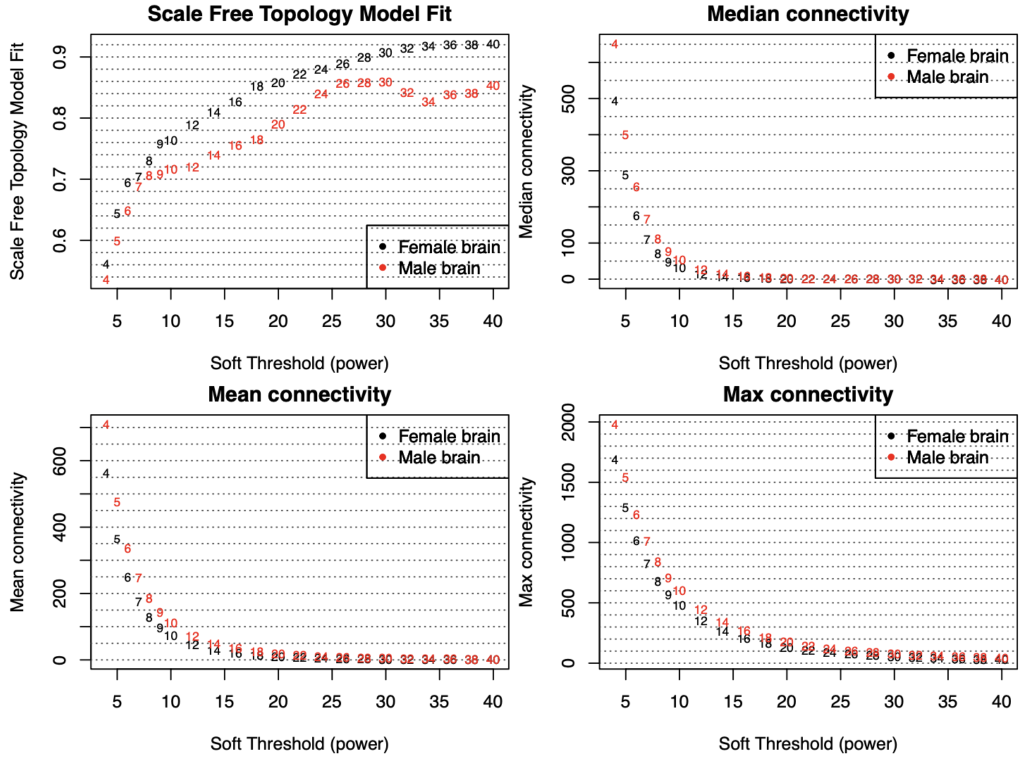

B.

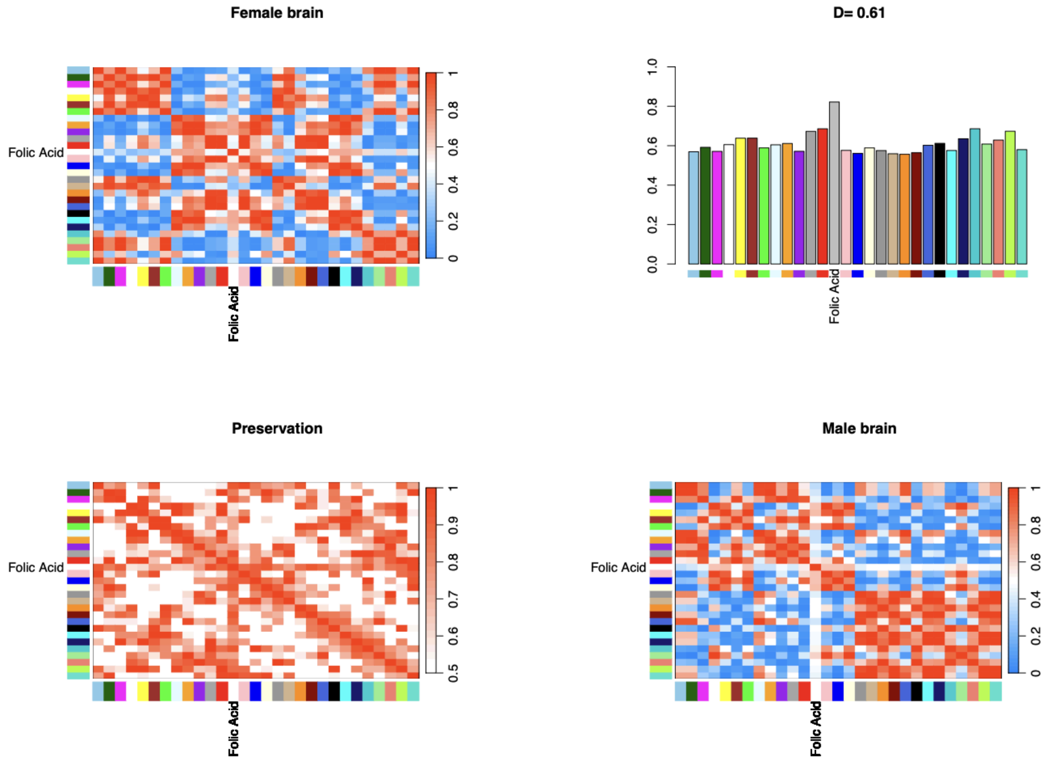

Fig. S8: Folic Acid sex-consensus network for brains. A) Network topology analysis. A soft-thresholding power of 28 was selected to construct scale-free networks. **B)** Module preservation statistics. The overall preservation of the eigengene networks is denoted by D=0.61.

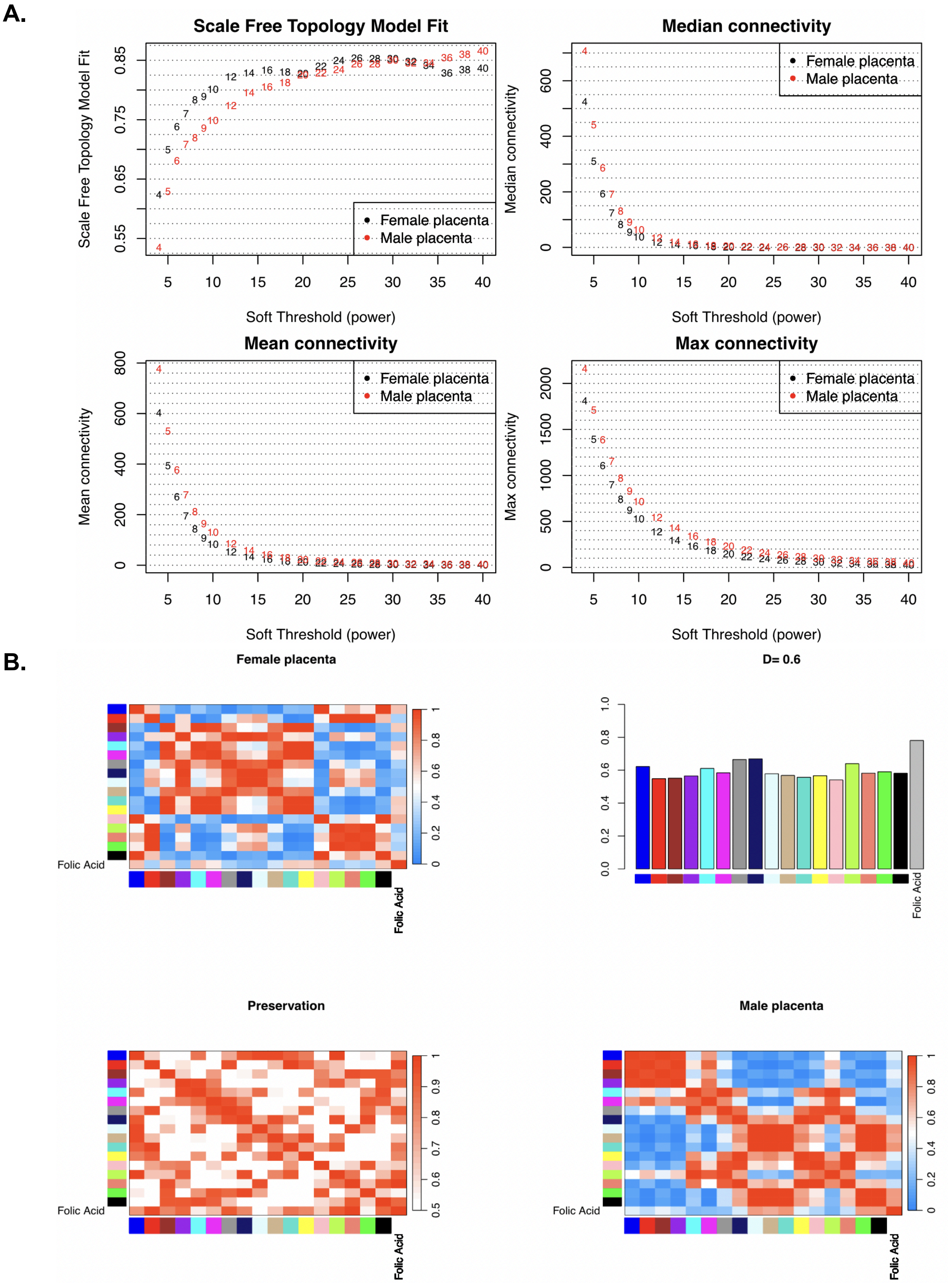

Fig. S9: Folic Acid sex-consensus networks for placenta. A) Network topology analysis. A soft-thresholding power of 28 was selected to construct scale-free networks **B)** Module preservation statistics. The overall preservation of the eigengene networks is denoted by D=0.6.

A.

B.

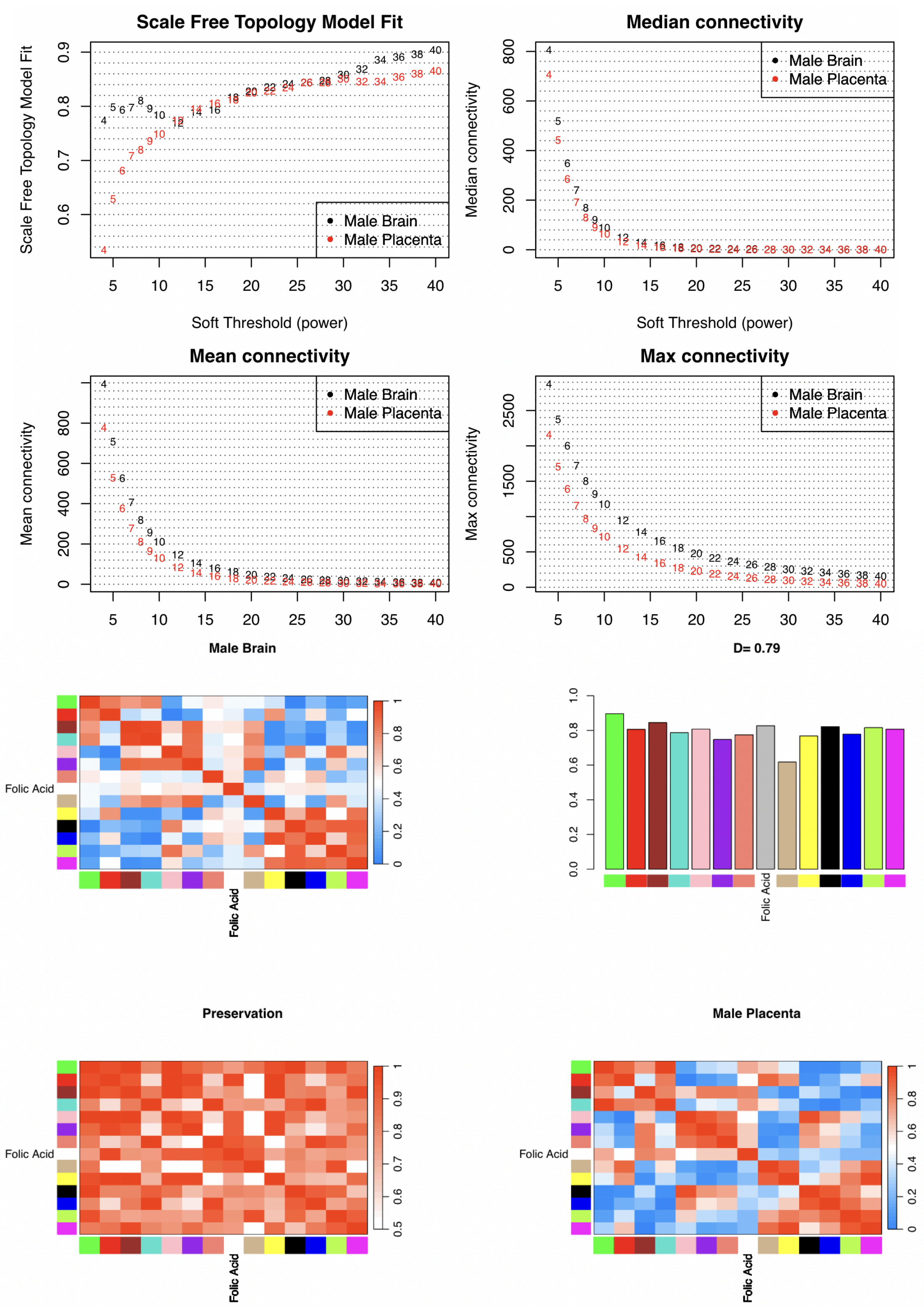

Fig. S10: Folic Acid tissue-consensus networks for males. A) Network topology analysis. A soft-thresholding power of 28 was selected to construct scale-free networks. **B)** Module preservation statistics. The overall preservation of the eigengene networks is denoted by D=0.79.

A.

B.

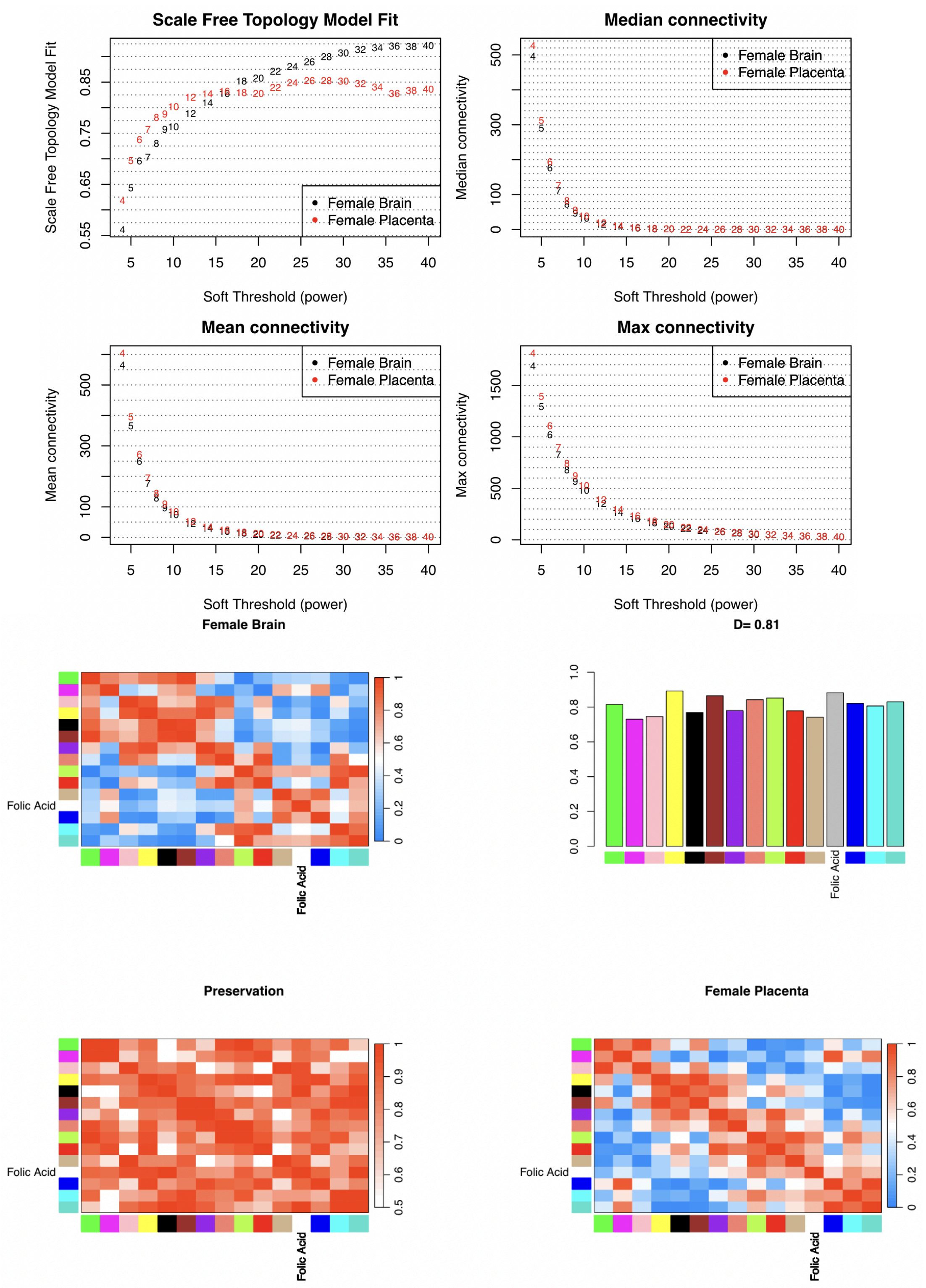

Fig. S11: Folic Acid tissue-consensus networks for females. A) Network topology analysis. A soft-thresholding power of 24 was selected to construct scale-free networks. **B)** Module preservation statistics. The overall preservation of the eigengene networks is denoted by D=0.81.

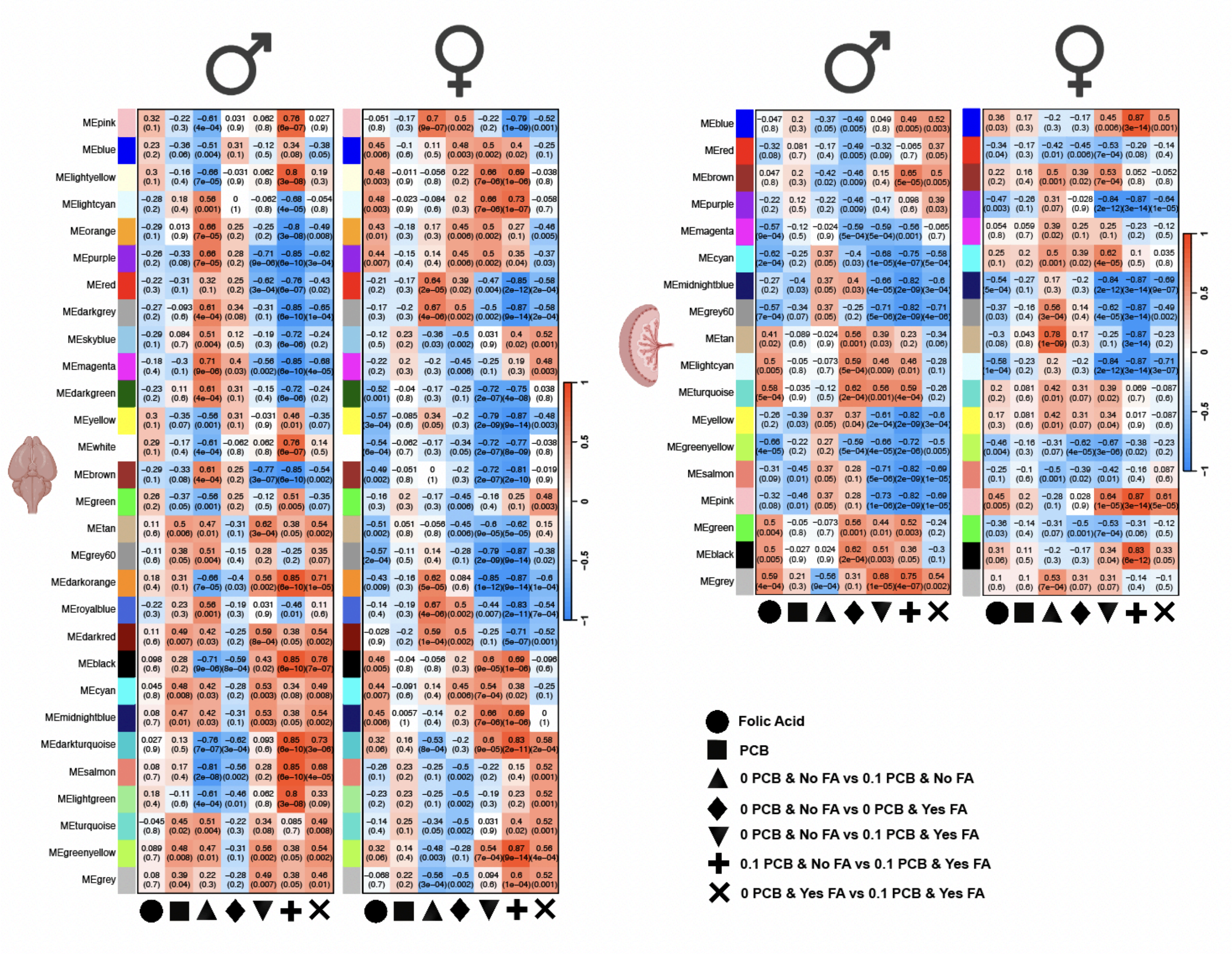

Fig. S12: Additional pairwise comparisons for sex-consensus folic acid networks. This is an expanded version of Figure 6 but with all the pairwise comparisons.

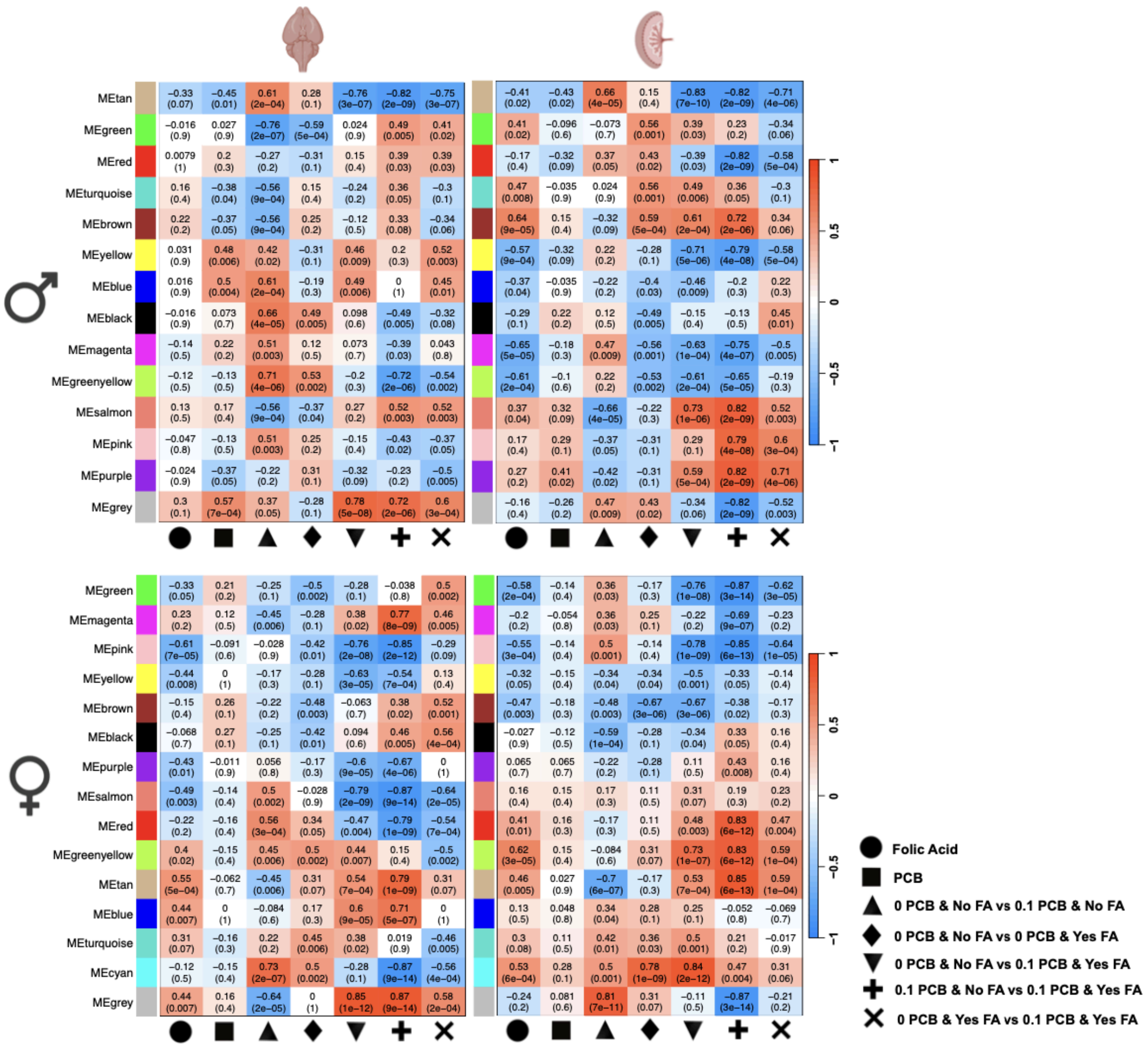

Fig. S13: Additional pairwise comparisons for tissue-consensus folic acid networks.
This is an expanded version of figure 7 but with all the pairwise comparisons.
